# Suppressive Immune Response of Poly(sarcosine) Conjugated Proteins: PSARylation

**DOI:** 10.1021/acs.bioconjchem.6c00159

**Published:** 2026-07-15

**Authors:** Noritomo Tsuda, Yota Okuno, Akari Yasuda, Mitsuru Ando, Yasuhiko Iwasaki

**Affiliations:** † Graduated School of Science and Engineering, 12860 Kansai University, 3-3-35 Yamate-cho, Suita-shi, Osaka 564-8680, Japan; ‡ Department of Chemistry and Materials Engineering, Kansai University, 3-3-35 Yamate-cho, Suita-shi, Osaka 564-8680, Japan; § Organization for Research & Development of Innovative Science & Technology, Kansai University, 3-3-35 Yamate-cho, Suita-shi, Osaka 564-8680, Japan; ∥ Institute for Life and Medical Sciences, 12918 Kyoto University, 53 Shogoin Kawahara-cho, Sakyo-ku, Kyoto 606-8507, Japan

## Abstract

Polymer conjugation is a widely used strategy to improve the pharmacokinetics of protein therapeutics by extending their half-life and protecting them from degradation. Poly­(ethylene glycol) (PEG) is the most commonly used polymer for this purpose. However, its use can trigger unwanted immune responses, leading to the production of anti-PEG antibodies that compromise efficacy. This issue has driven the search for alternative polymers, with poly­(sarcosine) (PSAR), derived from an endogenous amino acid, emerging as a promising candidate. Despite this potential, the immunogenicity of PSAR–protein conjugates (PSARylation) has not been systematically compared with that of PEGylated counterparts. In this report, we show that conjugating a model protein antigen to high-molecular-weight PSAR more effectively suppresses immune responses against both the protein and the polymer than PEG. Although polymer conjugation has traditionally been viewed as a means of steric “masking” of a protein, our results demonstrate that the chemical nature of the polymer is a critical, independent factor, as PSAR conjugates elicited lower antibody production than PEG conjugates of identical hydrodynamic size. This study highlights polymer selection as an important design parameter for minimizing immunogenicity in next-generation protein therapeutics. Polymers derived from biological building blocks, such as PSAR, offer a promising route to developing safer, more effective biopharmaceuticals with better tolerance and reduced risk of adverse immune reactions.

## Graphical abstract

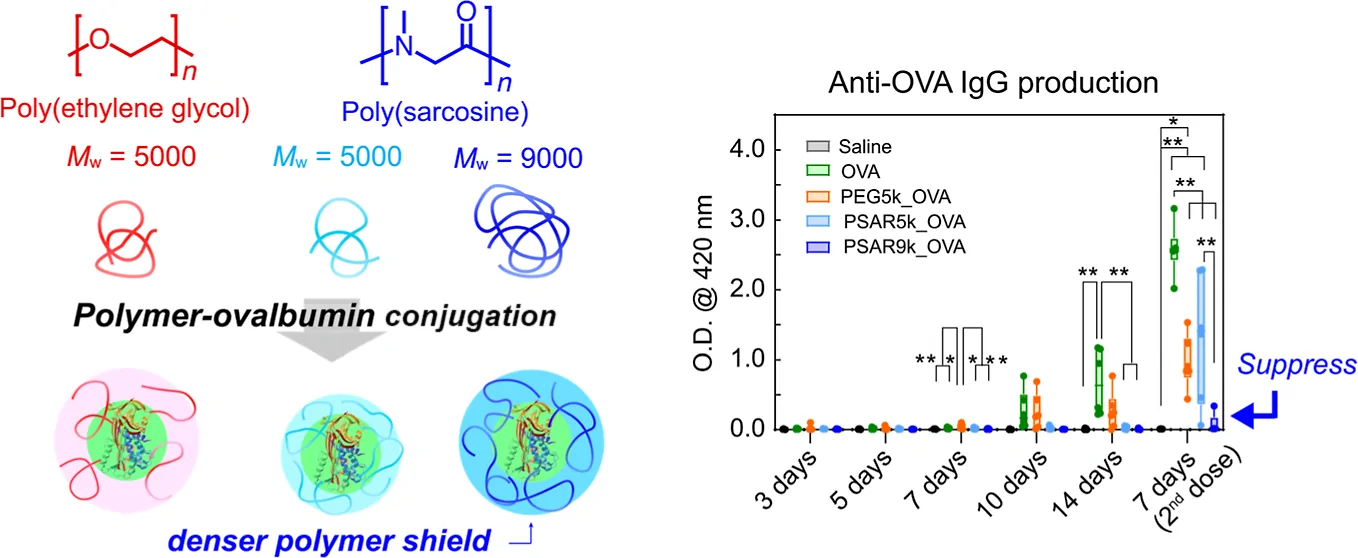

## 1 Introduction

Protein-based drugs, or biologics, exhibit potent therapeutic efficacy against various diseases, including diabetes, rheumatism, cancer, and hepatitis C.^1^ According to the Therapeutic Protein Database2 (THPdb2), more than 894 therapeutic proteins have been approved by the U.S. Food and Drug Administration (FDA).^2^ Despite their excellent pharmacological activity, protein drugs face several inherent limitations, including short circulation half-lives, susceptibility to enzymatic degradation, and immunogenicity.^3^ Covalent conjugation of polymers to a target protein has therefore emerged as a promising strategy to overcome these limitations. In 1977, Davis et al. first demonstrated that conjugating methoxy poly­(ethylene glycol) (mPEG) to bovine serum albumin (BSA) prolonged its blood circulation time by suppressing immunogenicity.^4^ The enhanced circulation time was subsequently attributed, in part, to the increased hydrodynamic size of the conjugate, which reduces renal clearance.^5,6^ Furthermore, polymer conjugation can shield proteins from proteolysis and phagocytosis, further extending their half-life in the bloodstream.^7^ Leveraging these advantages, 31 polymer–protein conjugates have received FDA approval.^8,9^

An ideal polymer for protein conjugation should be hydrophilic, have a high molecular weight, and exhibit low immunogenicity.^10^ Poly­(ethylene glycol) (PEG) fulfills many of these criteria and is widely regarded as a “stealth” polymer because of its hydrophilic, nonionic, and nonfouling properties.^7^ The ability to synthesize PEG with relatively narrow molecular weight distributions established it as the benchmark polymer for protein conjugation, a process commonly referred to as “PEGylation”.^9^ Over the past five decades, extensive studies have meticulously investigated how conjugation strategies, attachment sites, polymer-to-protein ratios, and the chemical characteristics of PEG (e.g., terminal groups, molecular weight, and architecture such as linear vs branched) affect protein activity, pharmacokinetics, and immunogenicity.^11–14^ Despite these efforts, the induction of anti-PEG antibodies remains a major limitation. Such antibodies accelerate the clearance of PEGylated proteins, thereby reducing their therapeutic efficacy, and may elicit allergic reactions in patients.^15–22^ It should be noted that these responses vary significantly depending on the route of administration. While PEGylated nanopharmaceuticals are utilized across diverse routes—including intravenous, subcutaneous, intramuscular, topical, and intravitreal pathways—the choice of route plays a critical role in the resulting immunogenicity.

Consequently, substantial effort has been devoted to identifying alternative polymers that mitigate PEG-associated immune responses. Among the candidates explored, poly­(sarcosine) (PSAR), or poly­(*N*-methyl glycine), has attracted considerable interest because of its nonionic, nonfouling, and highly hydrophilic characteristics, which closely resemble those of PEG.^23–27^ PSAR also exhibits excellent biocompatibility because its monomer, sarcosine, is an endogenous amino acid, and the polymer is highly resistant to proteolytic degradation.^28^ A key advantage of PSAR is its precise synthetic control: living ring-opening polymerization of sarcosine *N*-carboxyanhydride enables narrow molecular weight dispersity (*D̵*_M_ < 1.1) and tunable degrees of polymerization of up to 4000.^24,29,30^

Based on these properties, PSAR has been investigated as a PEG alternative, particularly in nanoparticle-based drug delivery systems. The immunogenicity of PSAR-modified liposomes and polymersomes has been examined in several studies.^31–35^ Notably, Haas et al. directly compared PEG- and PSAR-coated liposomes for mRNA delivery and reported a reduced immune response for PSAR-coated systems.^36^ Similarly, Ueda et al. demonstrated that PSAR-functionalized liposomes retained prolonged circulation times after repeated administration, effectively suppressing the accelerated blood clearance phenomenon observed for PEGylated counterparts.^37,38^ Despite these advances, studies on the immunogenicity of PSAR–protein conjugates remain limited. Currently, only one study reported by Lu et al. has shown that a PSAR–interferon-α2b conjugate suppressed anti-IFN IgG production more effectively than its PEGylated analog 3 weeks after initial administration.^39^ However, a detailed understanding of the immune response to PSAR, along with direct comparisons with PEG in protein conjugates, is lacking. Furthermore, the effects of PSAR conjugation on the protein’s structural integrity have not been systematically examined.

In this study, we specifically focused on comparing the humoral immune response elicited by subcutaneous (SC) injection. The SC route was strategically selected as it represents a more stringent and sensitive model for evaluating “immunological shielding” efficacy. Furthermore, the SC route is of paramount clinical importance for the self-administration of modern biologics. Herein, we report the synthesis of poly­(sarcosine)-conjugated bovine serum albumin (PSAR–BSA) at varying polymer-to-protein ratios and evaluate the effects on secondary structure, enzymatic activity, and proteolysis resistance. In addition, three different ovalbumin (OVA) conjugates were prepared: one methoxy-terminated PEG conjugate (mPEG–OVA) and two methoxy-terminated PSAR conjugates (mPSAR–OVA) with different molecular weights. The mPSAR polymers were strategically selected such that one matched the radius of gyration of mPEG, whereas the other yielded a conjugate with a hydrodynamic radius comparable to that of the mPEG–OVA conjugate. These conjugates were administered subcutaneously to mice, and the production of anti-OVA, anti-mPEG, and anti-mPSAR antibodies was monitored. A booster immunization was given 2 weeks after the primary immunization to assess the memory immune response. Our findings reveal that conjugation with higher-molecular-weight mPSAR shields OVA from immune recognition, leading to a pronounced suppression of anti-OVA IgM and IgG production compared with the production with PEGylation. To the best of our knowledge, this study represents the first direct comparison of immune responses in mice elicited by a PSAR-modified protein and its PEGylated counterpart.

## 2 Results and Discussion

### 2.1 Synthesis of Poly­(sarcosine) and Conjugation to Bovine Serum Albumin

Poly­(sarcosine) (PSAR) was synthesized via ring-opening polymerization of sarcosine *N*-carboxyanhydride (NCA), as shown in Figure 1a.^40^ Covalent conjugation to bovine serum albumin (BSA) required PSAR bearing a *C*-terminal carboxylic acid group. Initial attempts to polymerize sarcosine did not yield a unimodal polymer, likely because of the poor solubility of sarcosine in dimethylformamide (DMF). This limitation was addressed by developing an in situ initiation strategy based on partial hydrolysis of SAR-NCA using a small amount of water. Water was injected into a SAR-NCA solution in DMF (molar ratio of [water]/[SAR-NCA] = 9.2:1000), and the mixture was stirred under an argon atmosphere at 25 °C for 24 h. This strategy successfully yielded a PSAR homopolymer with a free *C*-terminal carboxylic acid and a unimodal molecular weight distribution. The *N*-terminus of the resulting PSAR was capped with hydrophilic glycolic acid to prevent self-condensation between polymer chains during protein conjugation.^41^ Size exclusion chromatography (SEC) analysis with PEG standard calibration yielded a weight-average molecular weight (*M*_w_) of 8.3 × 10^3^ g/mol, and a molecular weight dispersity (*M*_w_/*M*_n_) of 1.24 (Figure S2). Additionally, ^1^H nuclear magnetic resonance (^1^H NMR) spectroscopy confirmed the successful purification of the obtained PSAR (Figure S3).

**Figure 1:**
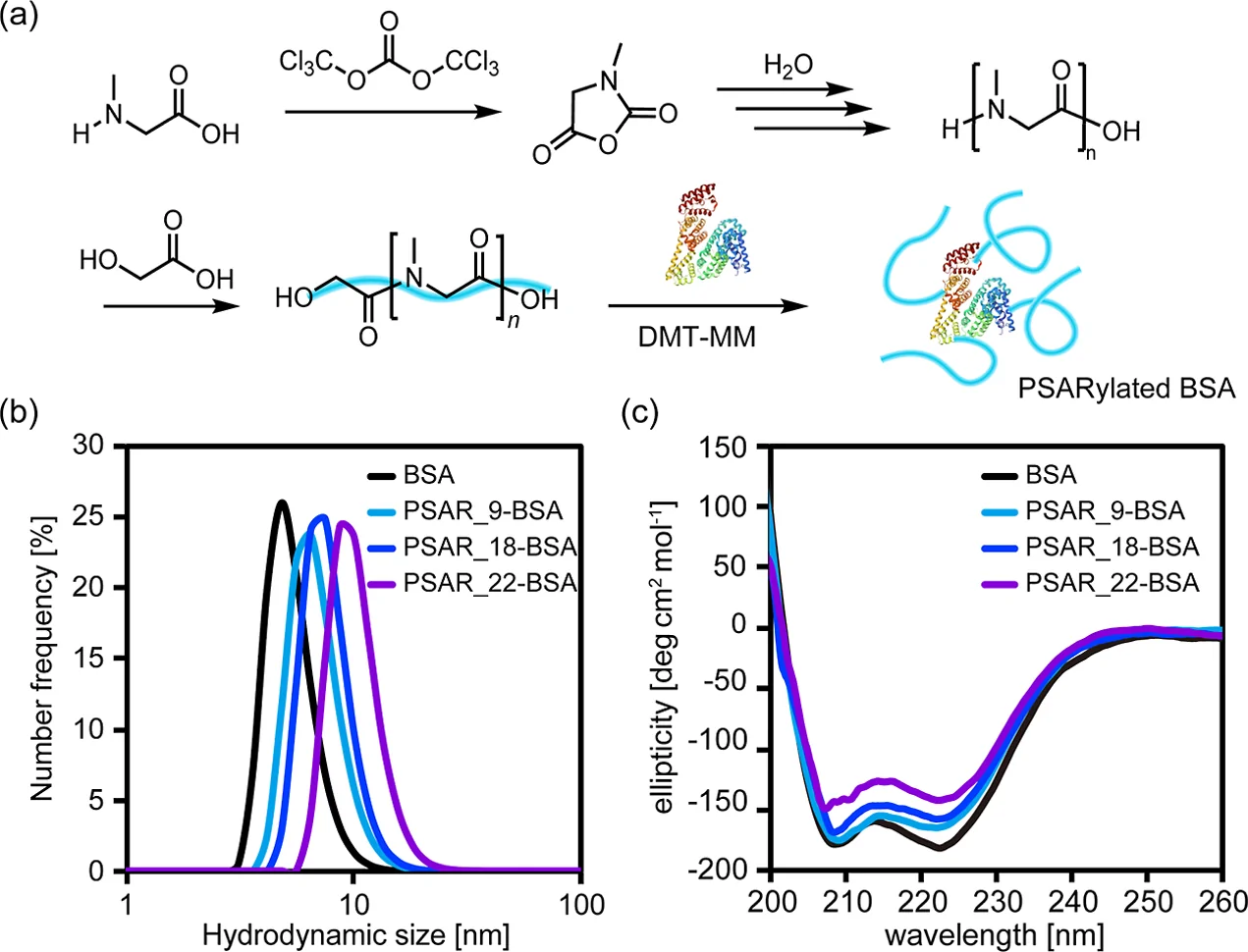
(a) Synthetic scheme of glycosylic acid-capped poly­(sarcosine) and poly­(sarcosine) conjugated bovine serum albumin (BSA). (b) The hydrodynamic size of PSARylated BSA with different numbers of PSAR chains was determined by dynamic light scattering. (c) Circular dichroism spectra of native BSA and PSARylated BSA.

Purified PSAR was conjugated to BSA using DMT-MM as a condensing agent, which promotes selective amide bond formation between the C-terminal carboxylic acid of PSAR and primary amino groups on the BSA surface (Figure 1a). Control over the number of PSAR chains attached per BSA molecule was achieved by varying the molar feed ratio of PSAR to BSA ([PSAR]/[BSA] = 15:1, 30:1, and 60:1), as summarized in Table 1. Sodium dodecyl sulfate-polyacrylamide gel electrophoresis (SDS-PAGE) confirmed an increase in the apparent molecular weight of BSA following PSAR conjugation (PSARylation) at all feed ratios (Figure S4). Quantitative analysis of residual free amino groups on BSA showed that the number of conjugated PSAR chains increased with increasing PSAR feed ratio (Table 1). The average number of conjugated PSAR chains per BSA molecule was 9, 18, and 22 for feed ratios of 15, 30, and 60, respectively. At the highest feed ratio, conjugation approached saturation, which is consistent with the approximately 30 primary amino groups reported to be solvent-accessible on the BSA surface.^42^

**Table 1:** Basic Physical Characteristics of PSARylated BSA

	[PSAR]/[BSA]			
	in feed	prepared	number-averaged hydrodynamic size/nm	the maintained secondary structure/%
BSA	-	-	6.5	100
PSAR_9-BSA	15	9.2 ± 0.2	7.0	92
PSAR_18-BSA	30	18 ± 0.2	7.8	87
PSAR_22-BSA	60	22 ± 0.5	10.3	78

### 2.2 Effect of PSAR Conjugation on the Secondary Structure and Enzymatic Activity of BSA

One of the key properties of the PSAR-BSA conjugates, hydrodynamic size, which is related to renal clearance, and residual enzymatic activity, which is related to therapeutic efficacy, were evaluated to assess whether PSARylation confers the expected benefits of polymer conjugation. Dynamic light scattering (DLS) analysis revealed a progressive increase in hydrodynamic size with the number of attached PSAR chains from 6.5 nm for native BSA to 7.3 nm (9 chains), 8.3 nm (18 chains), and 10.3 nm (22 chains) (Figure 1b and Table 1). The effect of PSAR conjugation on the secondary structure of BSA was examined by far-UV CD spectroscopy (Figure 1c). Analysis of the ellipticity at 222 nm indicated a modest reduction in α-helical content of BSA, decreasing to 92%, 87%, and 78% of the native structure for BSA modified with 9, 18, and 22 PSAR chains, respectively. These data indicate that PSARylation induces only limited structural perturbation, even for PSAR_22-BSA, in which nearly all accessible surface amino groups were modified.

Following confirmation that BSA retains structural integrity following conjugation, the esterase-like catalytic activity of PSARylated BSA was assessed by monitoring the hydrolysis of *p*-nitrophenyl acetate (*p*-NPA) to *p*-nitrophenol over a range of substrate concentrations (Figure S5).^43^ The kinetic parameters for native and PSARylated BSA were derived from Lineweaver–Burk analysis using the Michaelis–Menten model (Figure 2). As summarized in Table 2, the maximum catalytic velocity (*V*_max_) was essentially preserved after PSAR conjugation, remaining above 94% of the native activity even for PSAR_22-BSA, despite a slight decline that correlated with increasing numbers of attached chains. In contrast, the Michaelis constant (*K*_m_), which reflects substrate affinity, increased by 20–27%, with no clear dependence on the degree of PSARylation. A similar trend was observed for the catalytic efficiency (*K*_cat_/*K*_m_), which decreased by 20–25% and was also independent of the number of conjugated PSAR chains. Previous studies have identified Tyr411 and Arg410 within Sudlow’s site II as key residues involved in the rapid initial hydrolysis of *p*-NPA.^44^ The present results suggest that the amino groups in the vicinity of this active site are preferentially modified by PSAR even at low feed ratios. Such modification is likely to sterically hinder substrate access, leading to the observed increase in *K*_m_.

**Figure 2:**
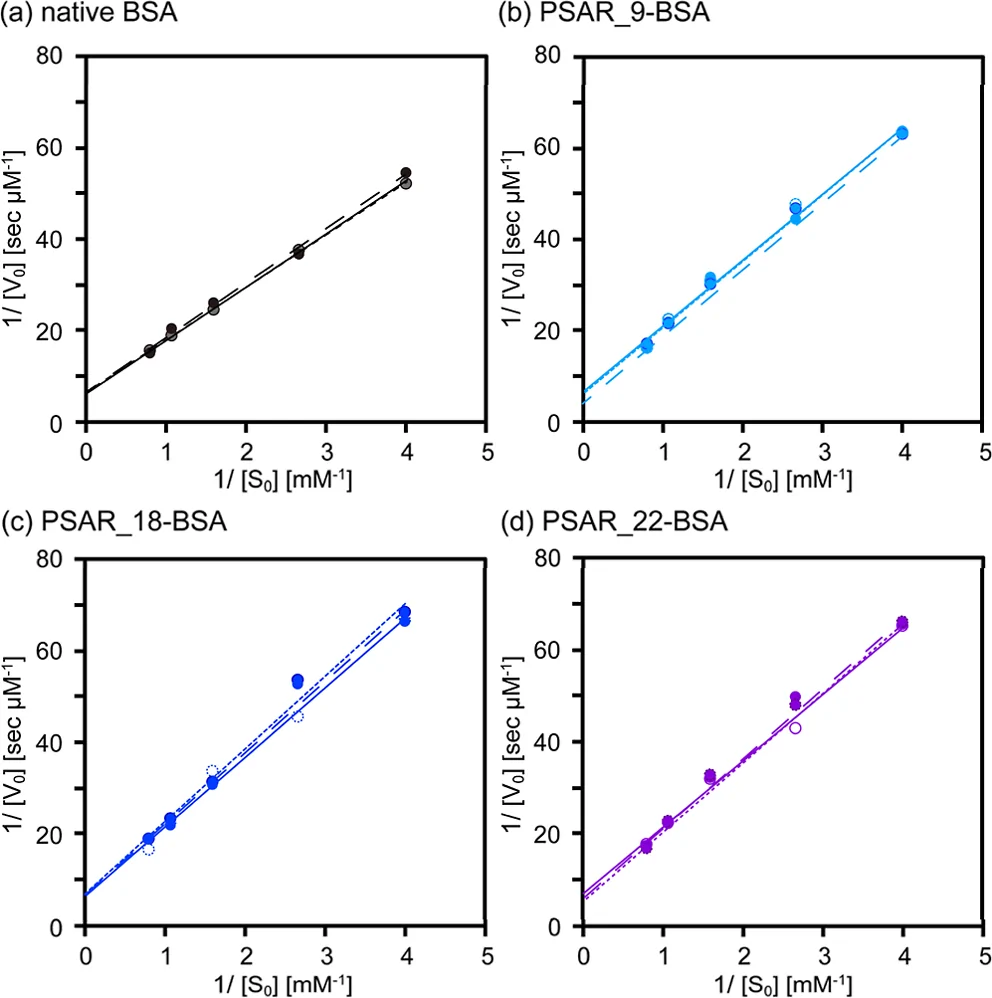
Lineweaver–Burk plots of BSA and PSARylated BSA at varying concentrations of *p*-NPA: (a) native BSA, (b) PSAR_9-BSA, (c) PSAR_18-BSA, and (d) PSAR_22-BSA (*n* = 3).

**Table 2:** Residual Esterase Activity of PSARylated BSA was Analyzed by the Michaelis–Menten Model (*n* = 3)

	*V*_max_/μM s^–1^	*K*_m_/mM	*K*_cat_/*K*_m_/M^–1^ s^–1^
BSA	0.156 ± 0.006	1.83 ± 0.08	113.5 ± 0.2
PSAR_9-BSA	0.153 ± 0.003	2.21 ± 0.05	91.9 ± 0.4
PSAR_18-BSA	0.150 ± 0.004	2.32 ± 0.01	85.7 ± 0.2
PSAR_22-BSA	0.147 ± 0.002	2.09 ± 0.08	89.5 ± 0.2

### 2.3 Preparation and Characterization of OVA, PSARylated OVA, and PEGylated OVA

Following characterization of the general effects of PSAR conjugation on a model protein, its ability to mask proteins from immune recognition was examined, along with potential differences between immune responses elicited by PSAR and PEG. Ovalbumin (OVA) was selected as the model protein because of its well-documented high immunogenicity. The terminal group of the polymer can influence the immune response;^45,46^ therefore, a methoxy group was chosen for both polymers, enabling a direct comparison between PSAR and PEG. Methoxy-terminated PEG (mPEG) was selected as the reference material because it represents the most widely used PEG architecture in clinical applications. Two methoxy-terminated poly­(sarcosines) polymers (mPSAR) were synthesized via SAR-NCA polymerization using 2-methoxyethylamine as an initiator (Scheme S1). The *N*-terminal of each polymer was subsequently functionalized with succinic acid anhydride to facilitate conjugation to OVA (Figure 3a). SEC analysis showed a single, well-defined peak for each polymer, with *M*_w_ values of 4.9 × 10^3^ (mPSAR5k) and 9.0 × 10^3^ g/mol (mPSAR9k), as calibrated against PEG standards (Figure 3b). Additionally, both samples exhibited a very narrow *D̵*_M_ of 1.02 for PSAR5k and 1.05 for PSAR9k. The methoxy-terminated PSAR polymers were activated using disuccinimidyl carbonate. Following purification to remove residual condensation reagent to prevent interprotein cross-linking, the activated mPSAR5k and mPSAR9k were conjugated to OVA at a molar feed ratio of [mPSAR]/[OVA] = 50:1. For comparison, methoxy-terminated PEG with a molecular weight of 5 kDa (mPEG5k) was also conjugated to OVA under identical conditions. After removal of excess unreacted polymer, the resulting mPEG5k–OVA, mPSAR5k–OVA, and mPSAR9k–OVA were analyzed by SDS-PAGE, confirming an increase in apparent molecular weight and the absence of free polymer (Figure S6).

**Figure 3:**
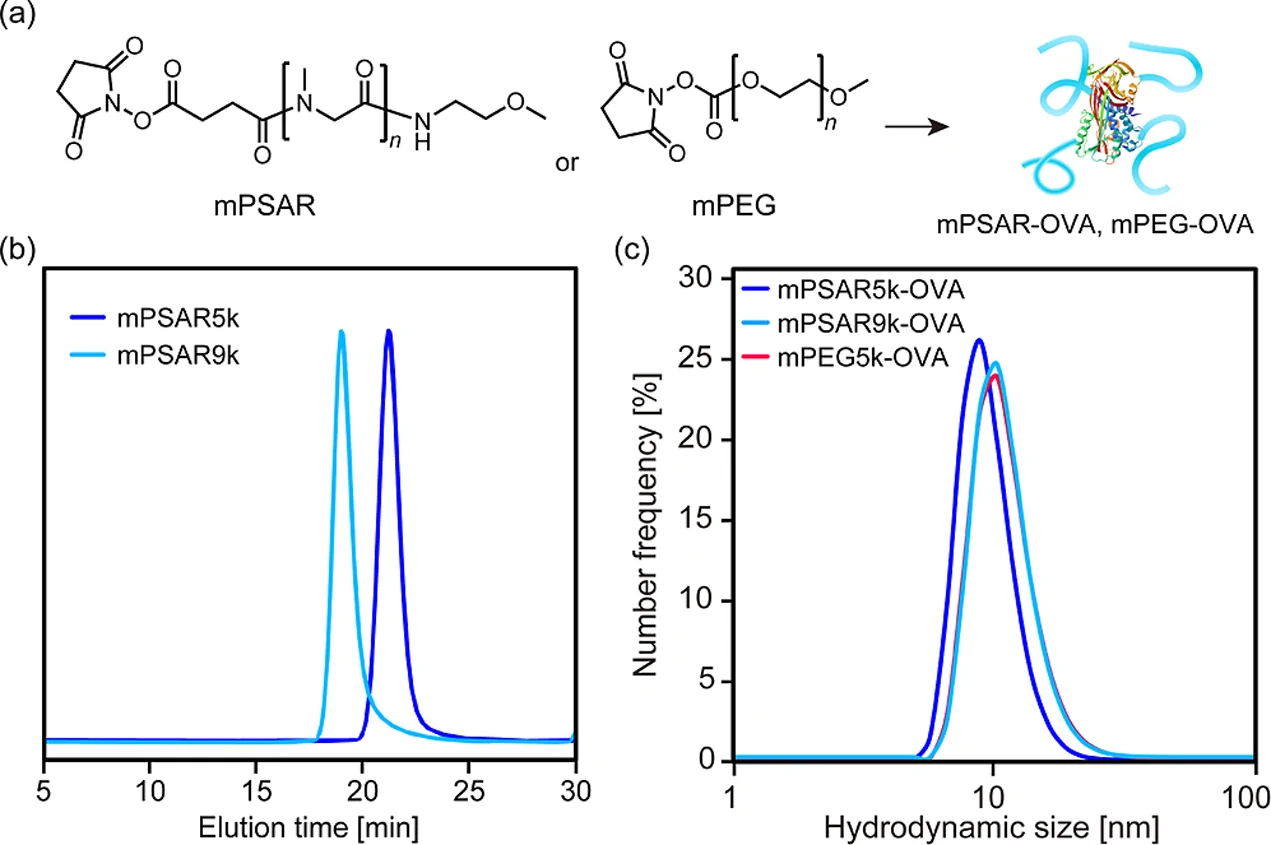
(a) Schematic showing the synthesis of mPSAR–OVA and mPEG–OVA. (b) Size-exclusion chromatograms of mPSAR5k and mPSAR9k, with the SE column calibrated using PEG standards. (c) The hydrodynamic size of mPSAR5k–OVA, mPSAR9k–OVA, and mPEG5k–OVA. mPSAR9k–OVA and mPEG5k–OVA exhibit similar size distributions.

Quantitative amino group analysis indicated that 21 polymer chains were attached per OVA molecule for all conjugates, mPSAR5k–OVA, mPSAR9k–OVA, and mPEG–OVA (Table 3). DLS measurements revealed that the hydrodynamic size of mPSAR5k–OVA was slightly smaller than that of mPEG5k–OVA, whereas mPSAR9k–OVA exhibited a hydrodynamic size comparable to mPEG5k–OVA (Figure 3c). This result suggests that mPSAR adopts a more compact conformation than mPEG at equivalent molecular weights. This carefully designed set of conjugates allows a direct comparison of immune responses between polymer conjugates of identical molecular weight (mPEG5k–OVA vs mPSAR5k–OVA) and conjugates with comparable hydrodynamic size (mPEG5k–OVA vs mPSAR9k–OVA). Finally, CD spectroscopy confirmed that secondary structure retention was approximately 90% (±3%) for all polymer-conjugated OVA samples (Figure 4), indicating minimal perturbation of the protein fold upon conjugation.

**Table 3:** Number of Conjugated Chains and the Hydrodynamic Size of PSARylated OVA (*n* = 3)

	[PSAR]/[BSA]		
	in feed	prepared	number-averaged hydrodynamic size/nm
OVA	-	-	4.7
mPSAR9k–OVA	50	21 ± 0.8	11.1
mPSAR5k–OVA	50	21 ± 2.1	8.7
mPEG5k_OVA	50	21 ± 0.8	11.0

**Figure 4:**
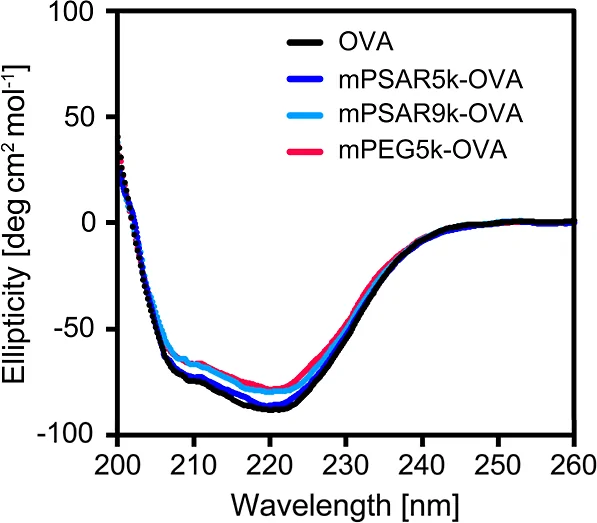
Far-UV circular dichroism spectra of native OVA, mPSAR–OVAs, and mPEG–OVA. The spectra demonstrate that the secondary structure of OVA was largely preserved following polymer conjugation, indicating that neither mPSAR nor mPEG significantly perturbs the protein fold of OVA.

### 2.4 Immune Response Against OVA, mPSAR Conjugated OVA, and mPEG Conjugated OVA

With the well-characterized conjugates in hand, we proceeded to evaluate the immune response in mice. Saline solutions of native OVA (positive control), mPEG5k–OVA, mPSAR5k–OVA, mPSAR9k–OVA, or saline (negative control) were administered by subcutaneous injection into BALB/c mice (6 week-old females). Here, the subcutaneous route was selected not merely as a single administration pathway, but to compare the shielding efficacy of PSARylation with PEGylation in the most immunogenic route.^47^ The dose was standardized to 0.15 μg of OVA in 50 μL based on BCA assay, a concentration determined from preliminary studies to be optimal for assessing immune responses and consistent with previous reports.^37^ Serum samples were collected on days 3, 5, 7, 10, and 14 after the primary immunization (Figure 5a). A booster dose was administered under identical conditions on day 15, followed by collection of a final serum sample on day 22 (7 days postbooster dose). Antibody titers against OVA, mPEG, and mPSAR (IgM and IgG) in each serum sample were quantified by enzyme-linked immunosorbent assay (ELISA).

**Figure 5:**
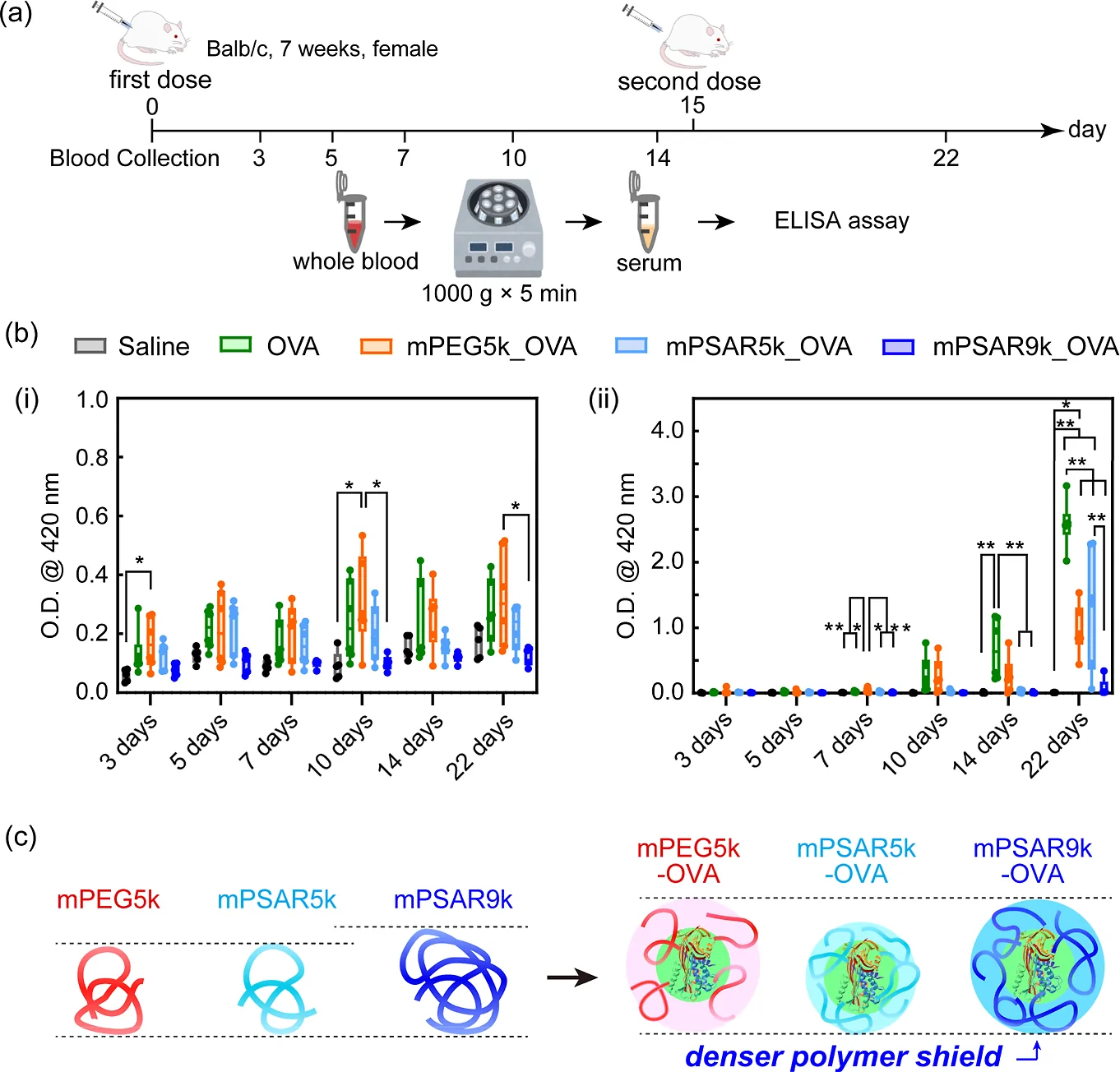
(a) Experimental scheme used to evaluate the immune responses in mice. (b) ELISA absorbance measured from sera diluted 90-fold for (i) anti-OVA IgM, (ii) anti-OVA IgG. Each dot represents an individual animal, and error bars indicate the standard deviation (*n* = 5 or 6 independent experiments). Adjusted *P* values: *P < 0.05 and **P < 0.01 by the Tukey–Kramer method. (c) Schematic illustration of the hydrodynamic sizes of free polymers and their corresponding ovalbumin (OVA) conjugates.

Focusing on the anti-OVA response, IgM production was detected as early as day 3 in the native OVA, mPEG5k–OVA, and mPSAR5k–OVA groups and remained detectable through day 14 (Figure 5b­(i)). In contrast, anti-OVA IgM levels in the mPSAR9k–OVA group remained barely detectable and were comparable to the saline control throughout the study, including after the booster dose. Similarly, despite being reduced compared to native OVA (positive control), anti-OVA IgG was still detectable in the mPEG5k–OVA and mPSAR5k–OVA groups on days 10 and 14, whereas it remained negligible in the mPSAR9k–OVA group. These findings indicate that while all polymer modifications effectively suppress anti-OVA antibody levels, mPSAR9k-OVA notably exhibits a particularly pronounced inhibitory effect on antibody production (Figure 5b­(ii)).

These findings indicate that while all polymer modifications suppress OVA antigenicity, mPSAR9k exhibits a particularly pronounced inhibitory effect. Although the shorter mPSAR5k chains provided partial shielding, they were insufficient to fully mask the protein from immune recognition, likely allowing greater access by immune cells, complement components, and/or enzymes, resulting in higher antibody levels than observed with mPSAR9k–OVA. As evidence of this, despite the nearly identical hydrodynamic sizes of mPSAR9k–OVA and mPEG5k–OVA, as determined by DLS (Figure 3c), which might caused by the interaction between PSAR chains and the protein. This suggests that immunogenicity is influenced not only by the overall conjugate size but also by the chemical nature of the polymer. Although free mPSAR9k has a larger molecular weight than mPEG5k, both conjugates exhibited comparable hydrodynamic dimensions, indicating that mPSAR9k may form a denser or more compact polymer shell on the protein surface than mPEG5k (Figure 5c). Such a shell would likely shield antigenic epitopes more effectively and suppress immune recognition, an interpretation consistent with the greater chain stiffness (Kuhn length) reported for poly­(sarcosine) compared to PEG.^48^ Consequently, these results could demonstrate that PSARylation suppresses OVA immunogenicity more effectively than conventional PEGylation.

For the selective detection of antipolymer antibodies without interference from anti-OVA antibodies, ELISA plates (96-well) were prepared by covalently immobilizing the respective polymers without OVA. In the mPEG5k–OVA group, anti-mPEG IgM was detected by day 3, increased sharply by day 10, and remained elevated through day 14 (Figure 6a­(i)). At day 7 after the booster dose, anti-mPEG IgM levels were higher than after the first administration. In contrast, anti-mPEG IgM was barely detected in all other administered groups. Anti-mPEG IgG was detected mainly at 10 days after the initial administration and exclusively in the mPEG5k–OVA group, with a pronounced increase following the second administration (Figure 6a­(ii)). Anti-mPSAR IgM was detected from day 3 after the initial immunization, and these IgM levels peaked on day 5 and subsequently declined on days 7, 10, and 14 in both the mPSAR5k–OVA and mPSAR9k–OVA administered groups (Figure 6b­(i)). Anti-mPSAR IgG was detected only at low levels on days 10 and 14 (Figure 6b­(ii)). Seven days after the booster dose, anti-mPSAR IgG levels were significantly lower in the mPSAR9k–OVA group than in the mPSAR5k–OVA group.

**Figure 6:**
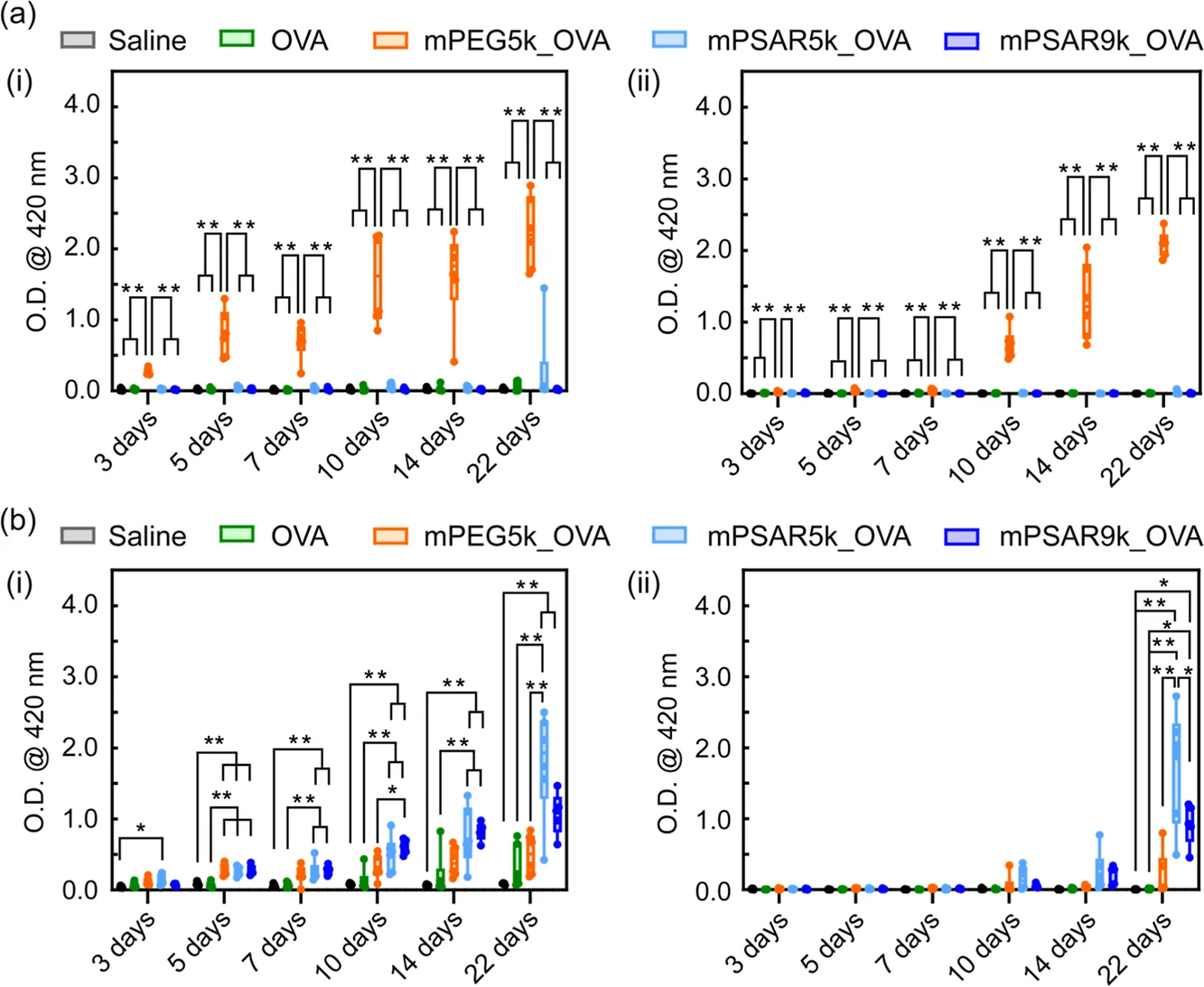
ELISA absorbance measured from sera diluted 90-fold for (a) (i) anti-PEG-IgM, (ii) anti-PEG-IgG and (b) (i) anti-PSAR-IgM, (ii) anti-PSAR-IgG. Each dot represents an individual animal, and error bars indicate the standard deviation (*n* = 5 or 6 independent experiments).

A more detailed analysis of antipolymer antibody production was obtained by fitting ELISA absorbance data taken from serially diluted serum samples to sigmoid curves (see experimental procedure). Antibody titers were defined as the serum dilution that yielded an absorbance (i.e., elicited immune response) exceeding the mean of the negative control group plus two standard deviations (Figures 7 and S7–S16 for raw data). This analysis revealed no significant difference in the anti-mPSAR titer between the mPSAR5k–OVA and mPSAR9k–OVA groups after the primary immunization. However, after the booster immunization, antibody levels were typically lower in the mPSAR9k–OVA group than in the mPSAR5k–OVA group. These results suggest that the longer mPSAR9k chains more effectively shield the OVA core, thereby reducing hapten-like behavior and suppressing the antipolymer antibody response. It should be noted that a direct comparison remains qualitative as the densities of mPEG and mPSAR covalently immobilized on the ELISA plates were not strictly determined. Nevertheless, mPSAR9k appeared to elicit lower antipolymer IgM and IgG titers compared to mPEG.

**Figure 7:**
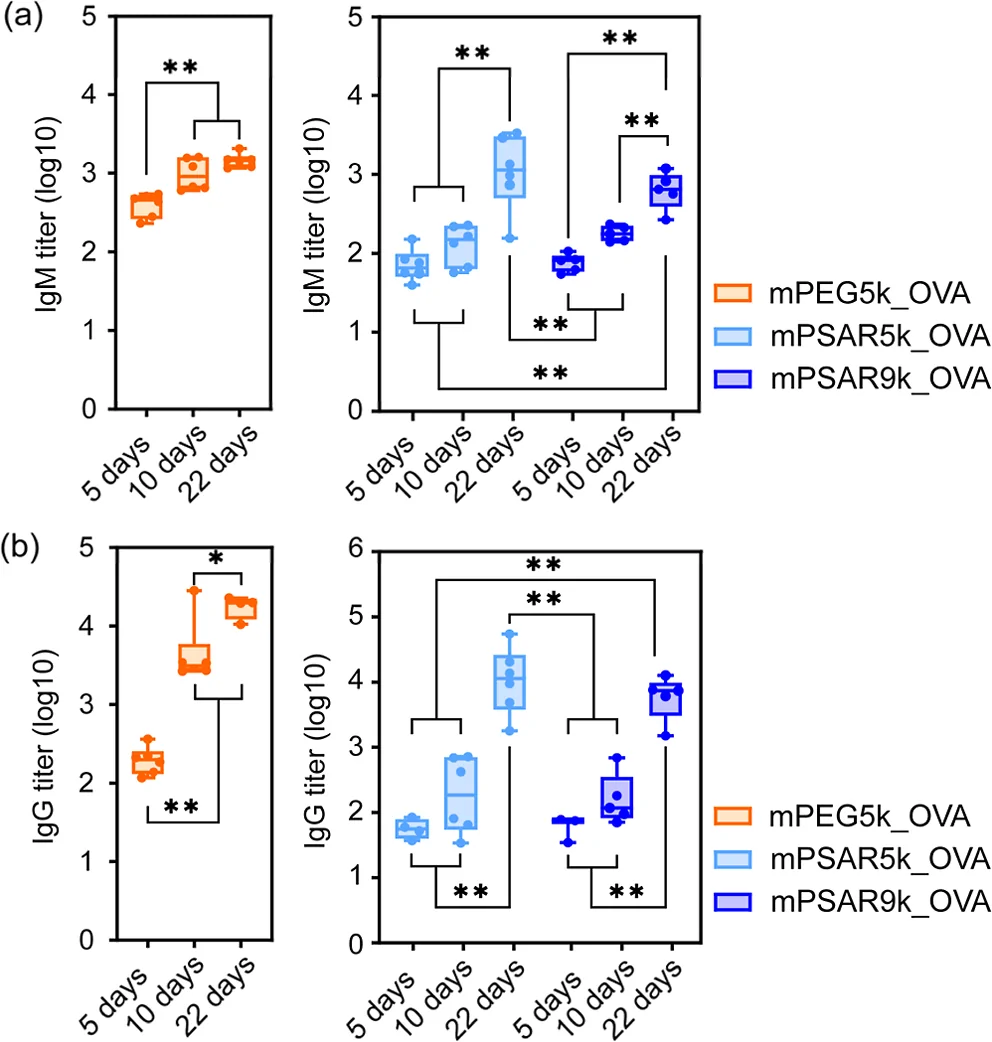
Serum dilution required to achieve an absorbance significantly higher than that of the saline control, as determined by four-parameter logistic regression: (a) antipolymer IgM and (b) antipolymer IgG. Each dot represents an individual animal, and the error bars indicate the standard deviation (*n* = 5 or 6 independent experiments). Adjusted *P* values: *P < 0.05 and **P < 0.01 by the Tukey–Kramer method. These data demonstrate that conjugation with mPSAR9k significantly suppresses the immune response against the conjugated polymer.

However, we acknowledge several limitations in the present study. Although extending the PSAR chain length from 5k to 9k demonstrated a noticeable reduction in antigenicity, parameters such as the molecular weight, the number of conjugated polymer chains, and the site-specificity of conjugation remain to be fully optimized. In clinically approved PEGylated proteins, the PEG molecular weight typically ranges from 5 to 40 kDa, and the number of conjugated chains is optimized between 1 and 17, depending on the specific requirements of the target protein.^8,9^ As an extension of this work, future studies employing actual therapeutic proteins should clearly elucidate whether optimizing the PSAR modification parameters—by referencing these established clinical formulations—can successfully achieve a simultaneous reduction in both antigenicity and immunogenicity compared to conventional PEGylation systems.

## 3 Conclusion

In this study, we demonstrated the successful application of poly­(sarcosine) (PSAR) for protein modification, a process termed “PSARylation.” Conjugation of PSAR to BSA at varying polymer-to-protein ratios showed that structural perturbations were modest and scaled with the degree of modification, with more than 80% of the native α-helical content retained. Furthermore, the esterase-like activity of BSA was largely preserved, with *V*_max_ remaining above 94% of that of the native protein. In addition, proteolysis resistance assays demonstrated that PSARylation significantly suppressed BSA degradation, with protection increasing as the number of conjugated PSAR chains increased.

Immunological evaluation using ovalbumin (OVA) as a model antigen further revealed that conjugation with high-molecular-weight mPSAR (mPSAR9k) effectively suppressed the host immune response against OVA. This masking effect proved more potent than that observed with a benchmark mPEG–OVA conjugate designed to have a comparable overall hydrodynamic size. Moreover, mPSAR conjugates showed a reduced tendency to elicit an antipolymer antibody response relative to PEGylated counterparts. These findings collectively indicate that PSARylation is an effective strategy for protecting proteins from degradation while preserving structural integrity and biological function, and for attenuating the immunogenicity of both the protein core and polymer shell. Increasing the molecular weight of the conjugated PSAR proved to be a key parameter in maximizing these immunological benefit.

PEGylation has been optimized over several decades through a wide range of modification strategies, including polymer molecular weights and architectures, and the present study does not claim an overall superiority of PSAR over PEG. Nonetheless, because PSAR is derived from the endogenous amino acid sarcosine, PSARylation represents a highly promising and potentially safer alternative for developing next-generation therapeutic protein conjugates.

## Supplementary Material



## Notes

All data needed to evaluate the conclusions in the paper are present in the paper and/or in the Supporting Information. Additional data related to this paper may be provided from the corresponding author (PDF)
