# Supplementary material for: Suppressive Immune Response of Poly(sarcosine) Conjugated Proteins: PSARylation
Source: Bioconjug Chem. 2026 Jul 15;37(8):1582–90. doi: 10.1021/acs.bioconjchem.6c00159 (PMC13495552; doi:10.1021/acs.bioconjchem.6c00159)
Supplement: Supplementary file 1 [file bc6c00159_si_001.pdf]

Supporting information

Suppressive Immune Response of Poly(sarcosine) Conjugated Proteins: PSARylation

Noritomo Tsuda[1], Yota Okuno[2,3]*, Akari Yasuda[1], Mitsuru Ando[4], Yasuhiko Iwasaki[2, 3]*

1. Graduated School of Science and Engineering, Kansai University, 3-3-35 Yamate-cho, Suita-shi, Osaka 564-8680 JAPAN
2. Department of Chemistry and Materials Engineering, Kansai University, 3-3-35 Yamate-cho, Suita-shi, Osaka 564-8680 JAPAN
3. Organization for Research & Development of Innovative Science & Technology, Kansai University, 3-3-35 Yamate-cho, Suita-shi, Osaka 564-8680 JAPAN
4. Institute for Life and Medical Sciences, Kyoto University, 53 Shogoin Kawahara-cho, Sakyo-ku, Kyoto 606-8507 JAPAN

Experimental Section

S1. Materials

Tetrahydrofuran (Super Dehydrated, Stabilizer Free) (purity > 99%), (R)-(+)-Limonene (purity > 99%), petroleum ether (purity > 90%), ethyl acetate (Super Dehydrated) (purity > 99%), Dimethylformamide (Super Dehydrated) (purity > 99%), pyridine (Super Dehydrated) (purity > 99%), diethyl ether (purity > 99%), *N,N*-diisopropylethylamine (purity > 97%) and methanol (purity > 99%) were purchased from Wako Pure Chemical Industries, Ltd, Japan. Sarcosine (purity > 98%), triphosgene (purity > 98%), 2-methoxyethylamine (purity > 98%), succinic anhydride (purity > 95%), di(*N*-succinimidyl) carbonate (purity > 98%), and glycolic acid (purity > 98%) were purchased from Tokyo Chemical Industry Co., Ltd, Japan. Ethyl 2-cyano-2-((dimethyliminio)(morpholino)methyloxyimino) acetate hexafluorophosphate (purity > 98%) and 4-(4,6-Dimethoxy-1,3,5-triazin-2-yl)-4-methylmorpholinium Chloride *n*-Hydrate were purchased from Watanabe Chemical Industry Co., Ltd, Japan. Albumin from bovine serum (purity > 96%), albumin from chicken egg white (purity > 98%), and poly(ethylene glycol) methyl ether (average *M*w=5k) were purchased from Sigma–Aldrich Co. LLC, USA. PD-10 prepacked column, Sephadex LH-20 and G-100 gel chromatogram supports and AKTA pure25 system were purchased from Cytiva, Japan. Dialysis membrane (MWCO 12–14 kDa, Spectrapore™) was purchased from Funakoshi Co., Ltd., Japan.

S2. Synthesis and preparations

S2-1. Synthesis of sarcosine N-carboxyanhydride (SAR-NCA)

Sarcosine (8.00 g, 89.8 mmol) was dissolved in 130 mL of dry tetrahydrofuran and limonene (19 mL, 117 mmol) was added. Triphosgene dissolved in 25 mL of dry tetrahydrofuran was added, and the mixture was stirred under nitrogen atmosphere at 68 °C for 4 h. After 4 h, the tetrahydrofuran and limonene were removed by an evaporator. The resulting solid was washed three times with petroleum ether and then dried in vacuo. The dried solid was sublimed at 80 °C with overnight.

The obtained solid was a white powder (6.44 g, 56.0 mmol, 62%). 1H NMR (400 MHz, chloroform-*d*): δ= 4.12 (s, 2H, N-CH_2-CO), 3.05 (s, 3H, -N-CH_3). (**Figure S1**)

S2-2. Synthesis of poly(sarcosine) for conjugation with bovine serum albumin

SAR-NCA (2.45 g, 21.3 mmol) was dissolved in 21 mL of dry dimethylformamide and water (3.5 μL, 193 μmol) was injected into the solution. The mixture was stirred under an argon atmosphere at room temperature for 24 h. Ethyl 2-cyano-2-((dimethyliminio)(morpholino)methyloxyimino)acetate hexafluorophosphate (COMU) (1.18 g, 2.76 μmol), and glycolic acid (137 μL, 2.70 μmol) were added to the solution, followed by addition of *N,N*-diisopropylethylamine (DIEA) (1.0 mL, 6.00 mmol). The mixture was stirred at room temperature for 48 h. Then the polymer solution was added to ethyl acetate and reprecipitated. The resulting solid was further purified by gel-filtration chromatography on Sephadex LH-20 eluted with methanol, using AKTA pure25 system with UV–Vis detector of 230 nm.

The obtained solid was a slightly yellow powder (1.27 g, 83%). $Đ_M$(GPC)=1.05. ^{1}H NMR (400 MHz, D_2O): δ=4.55–4.20 (broad, NH-CH_2-CO-), 3.15–2.98 (broad, -N-CH_3) (**Figure S2**)

S2-3. Preparation of PSAR conjugated bovine serum albumin

The C-terminal of poly(sarcosine) were activated by mixing poly(sarcosine) (1 eq.) with 4-(4,6-Dimethoxy-1,3,5-triazin-2-yl)-4-methylmorpholinium chloride (DMT-MM) (0.5 eq.) in phosphate buffered saline (PBS, pH=7.4) and stirring for 24 h at room temperature. The BSA solution of PBS was added to the activated polymer solution with the activated polymer to bovine serum albumin (BSA) ratio of 15:1, 30:1 and 60:1. The final BSA concentration was 0.15 mM, and the mixture was stirred at room temperature for 24 h. The conjugates were purified by gel-filtration chromatography on Sephadex G-100 eluted with PBS (pH=7.4). Chromatographic

separations were performed on an ÄKTA pure 25 system (Cytiva, Tokyo, Japan) equipped with conductivity and UV–Vis detectors. The system was controlled using UNICORN software (version 7.8, Cytiva). The fractions containing the conjugates were pooled, dialyzed against deionized water with using 12-14 kDa MWCO cellulose dialysis membrane, and then freeze-dried. The obtained sample were white powders.

S2-4. Synthesis of methoxy group terminated poly(sarcosine) (mPSAR) for conjugation with ovalbumin

SAR-NCA (3.00 g, 132 mmol) was dissolved in 26 mL of dry benzonitrile and 2-methoxyethylamine (25.0 μL, 290 μmol for mPSAR5k and 11.4 μL, 132 μmol for mPSAR9k) was injected into the solution. The mixture was stirred under an argon atmosphere at room temperature for 6 h. After 6 h, to introduce a carboxyl group at the *N*-terminal of the mPSAR, succinic anhydride (200 mg, 2.00 mmol for mPSAR5k and 210 mg, 2.10 mmol for mPSAR9k) was added and stirred under an argon atmosphere at room temperature for 48 h. Then the polymer solution was added to ethyl acetate and reprecipitated. The resulting solid was further purified by gel-filtration chromatography on Sephadex LH-20 eluted with methanol, yielding methoxy group terminated poly(sarcosine) (mPSAR).

The obtained solid was a white powder (1.67 g 87% for mPSAR5k and 1.82 g 96% for mPSAR9k). mPSAR5k: $Đ_M$(GPC)=1.02. ^{1}H NMR (400 MHz, D_2O): δ=4.51–4.25 (broad, -CH_2-CO-), 3.14–3.00 (broad, -N-CH_3). mPSAR9k: $Đ_M$(GPC)=1.05. ^{1}H NMR (400 MHz, D_2O): δ=4.55–4.20 (broad, -CH_2-CO-), 3.15–2.98 (broad, -N-CH_3).

S2-5. Preparation of methoxy group terminated poly(sarcosine) conjugated ovalbumin

As a typical procedure, methoxy group terminated poly(sarcosine) was dissolved in 4 mL of dry dimethylformamide, followed by adding di(*N*-succinimidyl) carbonate (1.5 eq.) and pyridine (2.3 eq.). The mixture was stirred under an argon atmosphere at room temperature overnight. The polymer solution was added to cold diethyl ether and reprecipitated. The obtained activated mPSAR was a white powder. Activated mPSAR was added in 4 mL of ovalbumin (OVA) solution (10 mg/mL) and stirred at room temperature for 4 h. The conjugates were purified by gel-filtration chromatography on Sephadex G-100 eluted with PBS (pH=7.4). The fractions containing the conjugates were pooled, desalted using a PD-10 column eluted with PBS (pH=7.4), and then freeze-dried. Chromatographic separations were performed on an ÄKTA pure 25 system (Cytiva, Tokyo, Japan) equipped with conductivity and UV–Vis detectors. The system was controlled using UNICORN software (version 7.8, Cytiva). The finally obtained mPSAR-OVA was a fluffy white powder.

S2-6. Preparation of methoxy group terminated poly(ethylene glycol) (mPEG) conjugated ovalbumin

As the same way as mPSAR-OVA, methoxy group terminated poly(ethylene glycol) (mPEG) was also activated by following way and conjugated to OVA. mPEG-OH (300 mg) was dissolved in 4.0 mL of dry acetonitrile, followed by adding di(*N*-succinimidyl) carbonate (230 mg, 0.90 mmol) and dry pyridine (108 μL, 1.34 mmol). The mixture was stirred under an argon atmosphere at room temperature overnight. Then the polymer solution was added to cold diethyl ether and reprecipitated. Activated mPEG (225 mg) was added in 4 mL of OVA solution (10 mg/mL) and stirred at room temperature for 4 h. The conjugates were purified by gel-filtration chromatography on Sephadex G-100 eluted with PBS (pH=7.4). The fractions containing the conjugates were pooled, desalted using a PD-10 column eluted with ultrapure water, and then freeze-dried. Chromatographic separations were performed on an ÄKTA pure 25 system (Cytiva, Tokyo, Japan) equipped with conductivity and UV–Vis detectors. The system was controlled using UNICORN software (version 7.8, Cytiva). The obtained sample was fluffy white powder.

S3. Evaluations

S3-1. Nuclear magnetic resonance spectroscopy

Nuclear magnetic resonance spectra were run with a JEOL ECZ400S 400MHz spectrometer to acquire ^{1}H NMR spectra. Chemical shifts (δ) are expressed in parts per million and are reported relative to solvent peaks or tetramethyl silane as an internal standard in ^{1}H NMR spectra.

S3-2. Gel permeation chromatography (GPC)

The apparent molecular weight and molecular weight distribution of polymer were assessed via gel permeation chromatography using Asahipak columns (GS-320 HQ and GS-520 HQ) with acetate buffer (0.1 M CH_3COONa, 0.3 M NaCl, 1.0 mM EDTA-2Na) as the solvent, at 25 °C (flow rate = 0.65 mL/min). HPLC system (JASCO, Japan) equipped with column oven (CO-2060Plus), pump (PU-2080Plus) and refractive index detector (RI-2031Plus). A calibration curve was created using 6 poly(ethylene glycol) standards [PEG: M_p=7.5 × 10^2 to 5.8 × 10^5 g/mol].

S3-3. SDS-polyacrylamide gel electrophoresis (SDS-PAGE)

Electrophoresis was carried out using 7.5 % polyacrylamide gel (ATTO, Tokyo, JP). Native protein and conjugates (3.0 mg/mL) were mixed with Ez Apply (ATTO, Tokyo, JP) and heated at 90 °C for 5 min. Ez Protein Ladder (ATTO, Tokyo, JP) was used as the marker. After electrophoresis, the gels were stained with Coomassie Brilliant Blue (ATTO, Tokyo, JP) to visualize proteins.

S3-4. Bicinchoninic acid assay

Native protein and conjugates were diluted to different concentrations ($1.0×10^{-1}$–$1.0×10^{-3}$ mg/mL), and protein quantification was carried out according to the instruction of Micro BCA™ Protein Assay Kit (Thermo Fisher Scientific, USA). The absorbance at 562 nm was measured by microplate reader (SH-9kLab, Corona Electric Co., Ltd., Japan). It should be noted that this analysis only evaluate the amount of protein, excluding poly(sarcosine) of poly(ethylene glycol).

S3-5. Dynamic light scattering

The hydrodynamic size (R_h) was characterized by dynamic light scattering (DLS) using a Zetasizer Nano ZS instrument (Malvern Instruments, UK). Native proteins and conjugates solutions (PBS with pH 7.4) was passed through a 0.25 μm filter. They were measured at a wavelength of 633 nm and a 173° detection angle at 25 °C.

S3-6. Circular dichroism spectroscopy

The circular dichroism (CD) spectra of BSA, OVA and their polymer conjugates solutions were measured between 200 and 260 nm with a scan rate of 100 nm/min at 25 °C using a J-1500 CD Spectrometer (JASCO, Tokyo, Japan). The obtained spectra for each sample were corrected by subtracting the spectrum of PBS (pH 7.4) as a blank. The measurements were performed in the wavelength range where the HT voltage remained under 600 V. For calculation of the mean residue ellipticity (MRE) [θ], the molecular weight of BSA was assumed to be 66 kDa, the average residue number of 583, and the molecular weight of OVA was assumed to be 45 kDa, the average residue number of 385. The mean residue ellipticity (MRE) [θ] was calculated using the following equations.

$$[\theta](\mathrm{deg}\cdot\mathrm{cm}^2/\mathrm{dmol}) = \frac{\mathrm{Observed}\theta \times \mathrm{M}}{10 \times \mathrm{n} \times \mathrm{C} \times \mathrm{l}}$$

where M is the molecular weight of protein, n is the average residue number of protein, C is the concentration of protein and l is the length of the sample cell.

S3-7. Esterase activity assay

To 1.5 mL of BSA or BSA-polymer conjugates PBS solutions (pH=8.0, 5.0×10^{-2} mg/mL as BSA eq.) was added 15 μL of p-nitrophenyl acetate (20-100 mM) as a substrate in acetonitrile. The esterase activity of BSA was evaluated by measuring the absorbance of released *p*-nitrophenolate ion as a product at 405 nm and 37 °C using a V-730 Spectrophotometer (JASCO, Tokyo, Japan). A molar extinction coefficient ε = 16302 $M^{-1}cm^{-1}$ (pH 8.0) was used in the reaction of *p*-nitrophenol. Initial reaction velocities (v_0) were calculated from the slope of the graph between 10 and 60 seconds. The kinetic parameters of BSA, including the maximum reaction velocity (V_{max}), Michaelis-Menten constant (K_m), and turnover number (K_{cat}), were calculated from the v0 using the following equations.

$$v_0 = \frac{V_{\max}[S]}{K_m + [S]}$$

$$V_{\max} = K_{cat}[E]$$

where [S] is the molar concentration of substrate (*p*-nitrophenyl acetate), and [E] is the total BSA concentration.

The kinetic parameters were used to create a Lineweaver–Burk plot.

S3-8. Animal experiment

The animal experiment protocol received approval from the Animal Experimentation Committee of Kansai University (Permit Number: 2422). BALB/c mice (4-week-old, female) were purchased from Japan SLC, Inc. (Shizuoka, Japan). Upon arrival, the mice were housed individually in cages and acclimated for 2 weeks prior to the experiments. For the control group, 50 μL of sterile saline was administered via subcutaneous injection. Other samples (OVA, mPEG-OVA, mPSAR5k-OVA, and mPSAR9k-OVA) were dissolved in 50 μL of saline and subcutaneously injected at a dose of 3 μg/shot (calculated based on OVA concentration), respectively. A second injection was administered 2 weeks after the first injection. Blood samples were collected from the tail vein on days 3, 5, 7, 10, and 14 after the first injection, and on day 7 after the second injection. To obtain serum, the collected blood was allowed to stand at room temperature for 10 min, followed by centrifugation at 1000 g for 5 min. The serum was stored at -80 °C until use. Throughout the experiment, mice were provided with a nonirradiated diet (5V5R, LabDiet., St. Louis, MO, USA). Two weeks after the final injection, the mice were euthanized by cervical dislocation following deep anesthesia with isoflurane. The level of anti-OVA IgM and IgG in serum was determined by enzyme-linked immunosorbent assay as described below.

S3-9. Enzyme-linked immunosorbent assay for anti-OVA antibodies

Maxisorp 96-well plates (Thermo Fisher Scientific, Waltham, USA) were coated with 10 μg/mL of OVA in ultrapure water by incubation at 4°C overnight. The coated plate was washed 5 times with 250 μL of wash solution, 50 mM Tris-buffer (pH = 7.4), 0.14 M NaCl, 0.05 % Tween-20. Then, 300 μL of blocking buffer was added and incubated for 1.5h. After washing, 50 μL of serum diluted to each concentration with blocking buffer (eBioscienceTM ELISA/ELISPOT Diluent (5X), Thermo Fisher Scientific, Waltham, USA) was added and incubated for 2h. After the plate was washed 6 times with 250 μL of wash solution, 50 μL of horseradish peroxidase (HRP)-conjugated goat anti-mouse IgM (1 mg/mL, Bethyl Laboratories, TX, USA) (1 : 10,000) or goat anti-mouse IgG (0.4 mg/mL, BioLegend, CA, USA) (1 : 10,000) was added and incubated for 1h. The plate was washed 5 times with 250 μL of wash solution and then added 50 μL of TMB solution (eBioscienceTM TMB Solution (1X), Thermo Fisher Scientific, Waltham, USA). After 15min, the reaction was stopped by adding 50μL of 1 M HCl. The absorbance was measured at 420 nm using a microplate reader (SH-9kLab, Corona Electric Co., Ltd., Japan). For titer analysis, the obtained relation between diluted concentration and absorbance was fitted by 4-parameters logistic regression for the following equation were performed using the '*drc*' package in R software (version 4.4.3).

$$f(x) = c + \frac{d - c}{1 + \exp\{b(\log x - \log e)\}}$$

Where *f(x)* represents the observed absorbance at a given plasma dilution x. The parameters are defined as follows: *b* is the Hill slope indicating the steepness of the curve; *c* and *d* are the lower and upper asymptotes, representing the minimum and maximum response levels, respectively; and *e* is the half-maximal effective concentration (EC_{50}), corresponding to the dilution value that produces a response halfway between *c* and *d*.

S3-10. Enzyme-linked immunosorbent assay for anti-polymers antibodies

Amino-group-bearing 96-well plates (MS-8698F, Sumitomo Bakelite Co., Ltd., Tokyo, Japan) were used to covalently immobilize succinimidyl-activated mPSAR or mPEG. 50 nmol of the polymer dissolved in 50 µL of saline was added to each well and incubated at 25 °C for 12 h. The plate was washed 5 times with 250 µL of wash solution (50 mM Tris-buffer (pH=7.4), 0.14 M NaCl, 0.05% Tween-20) to remove non-covalently bound polymers. Then, 300 µL of blocking buffer was added and incubated for 1.5 h. After washing the plate 5 times with wash solution, 50 µL of serum diluted to each concentration with blocking buffer (eBioscience™ ELISA/ELISPOT Diluent (5X), Thermo Fisher Scientific, Waltham, USA) was added and incubated for 2 h. The plate was washed 6 times with 250 µL of wash solution, and then 100 µL of horseradish peroxidase (HRP)-conjugated goat anti-mouse IgM (1 mg/mL, Bethyl Laboratories, TX, USA) (1:10,000) or goat anti-mouse IgG (0.4 mg/mL, BioLegend, CA, USA) (1:10,000) was added and incubated for 1 h. After the plate was washed 6 times with 250 µL of wash solution, 50 µL of TMB solution (eBioscience™ TMB Solution (1X), Thermo Fisher Scientific, Waltham, USA) was added. After 15 min, the reaction was stopped by adding 50 µL of 1 M HCl. The absorbance was measured at 420 nm using a microplate reader (SH-9kLab, Corona Electric Co., Ltd., Ibaraki, Japan).

S4. Safety statement

All experiments were carried out under standard laboratory safety protocols. Reagents, instruments, and workflows underwent routine safety evaluation and control. There was no unexpected or unusually high safety hazards were encountered.

Supplementary Figures

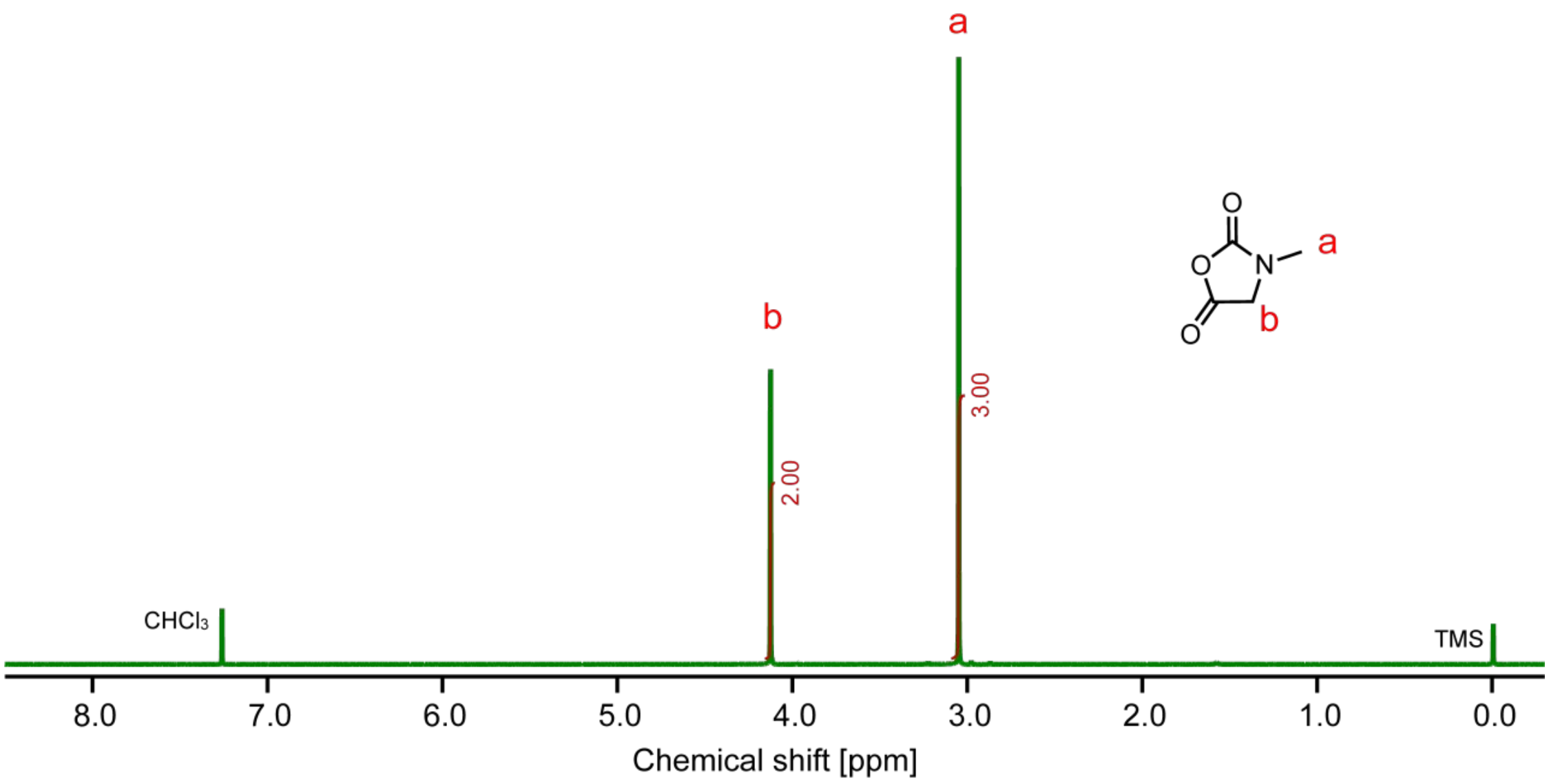

Figure S1. ^{1}H NMR spectrum of SAR-NCA in Chloroform-*d*. X-dimension is the chemical-shift (*δ*) and red values are relative integral values.

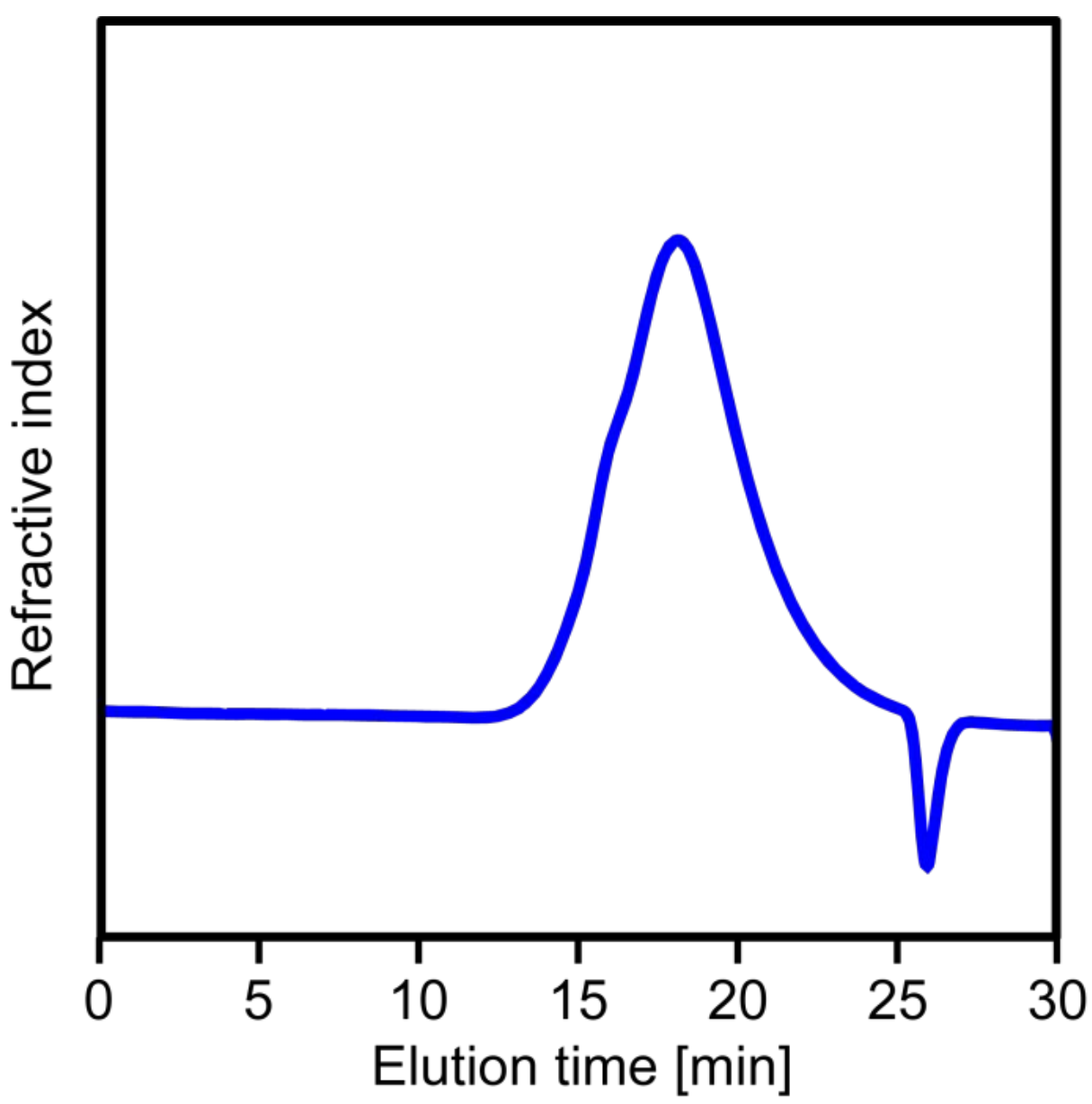

Figure S2. Size exclusion chromatograms of glycosylic acid terminated poly(sarcosine).

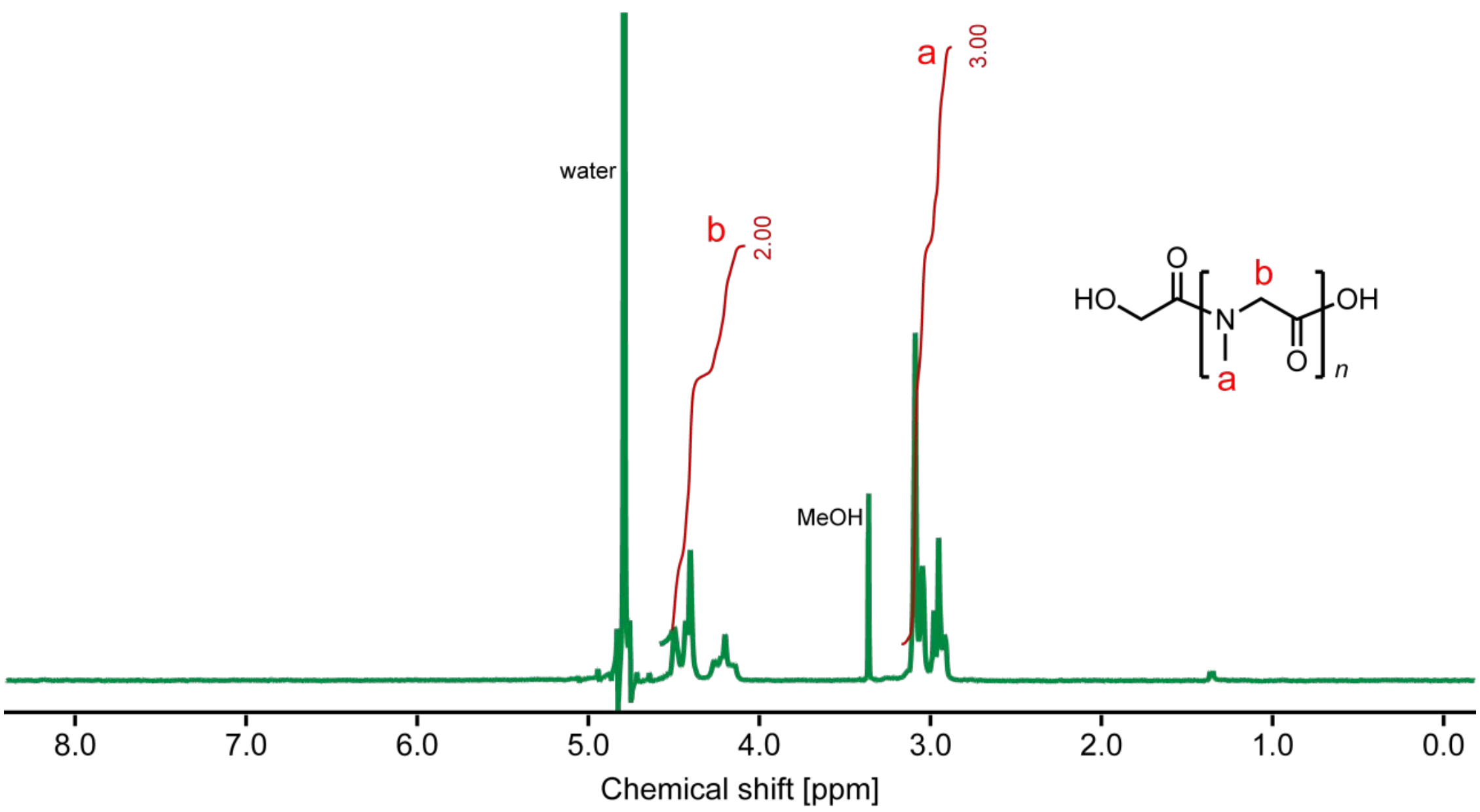

Figure S3. ^{1}H NMR spectrum of **polymer [1]** in D_2O. X-dimension is the chemical shift, and red values are relative integral values.

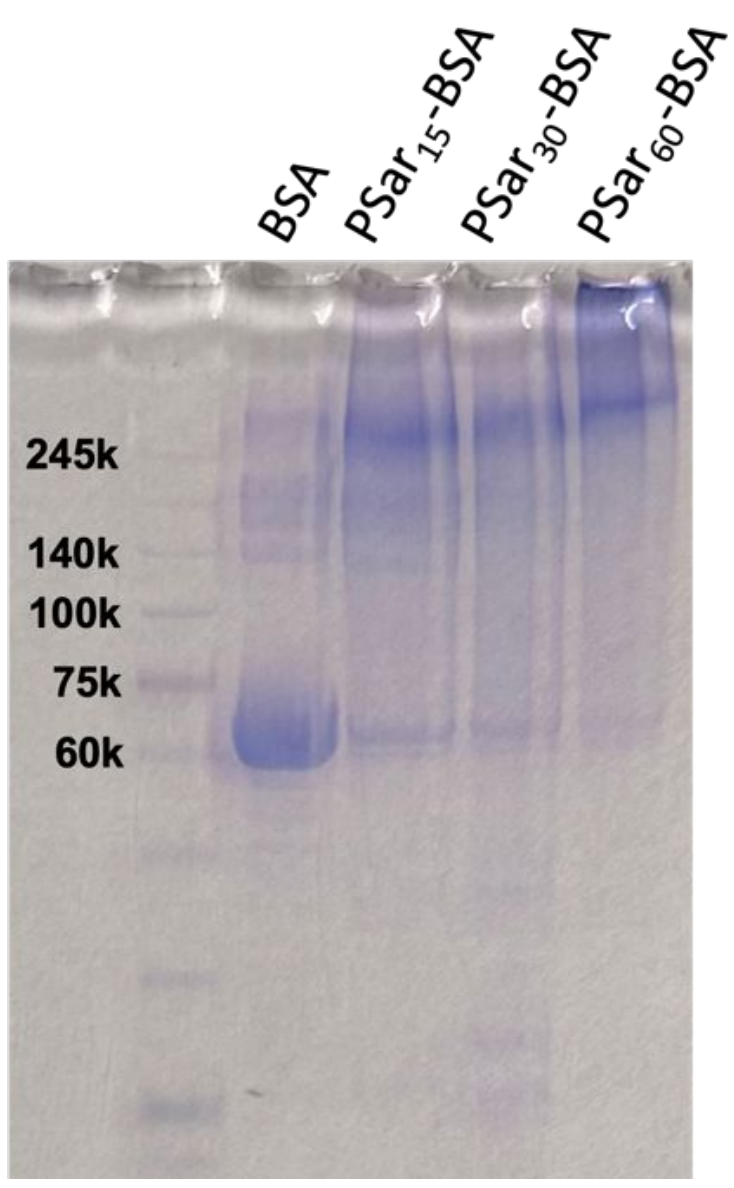

Figure S4. Molecular weights increase of BSA by the immobilization of PSar determined by SDS-PAGE.

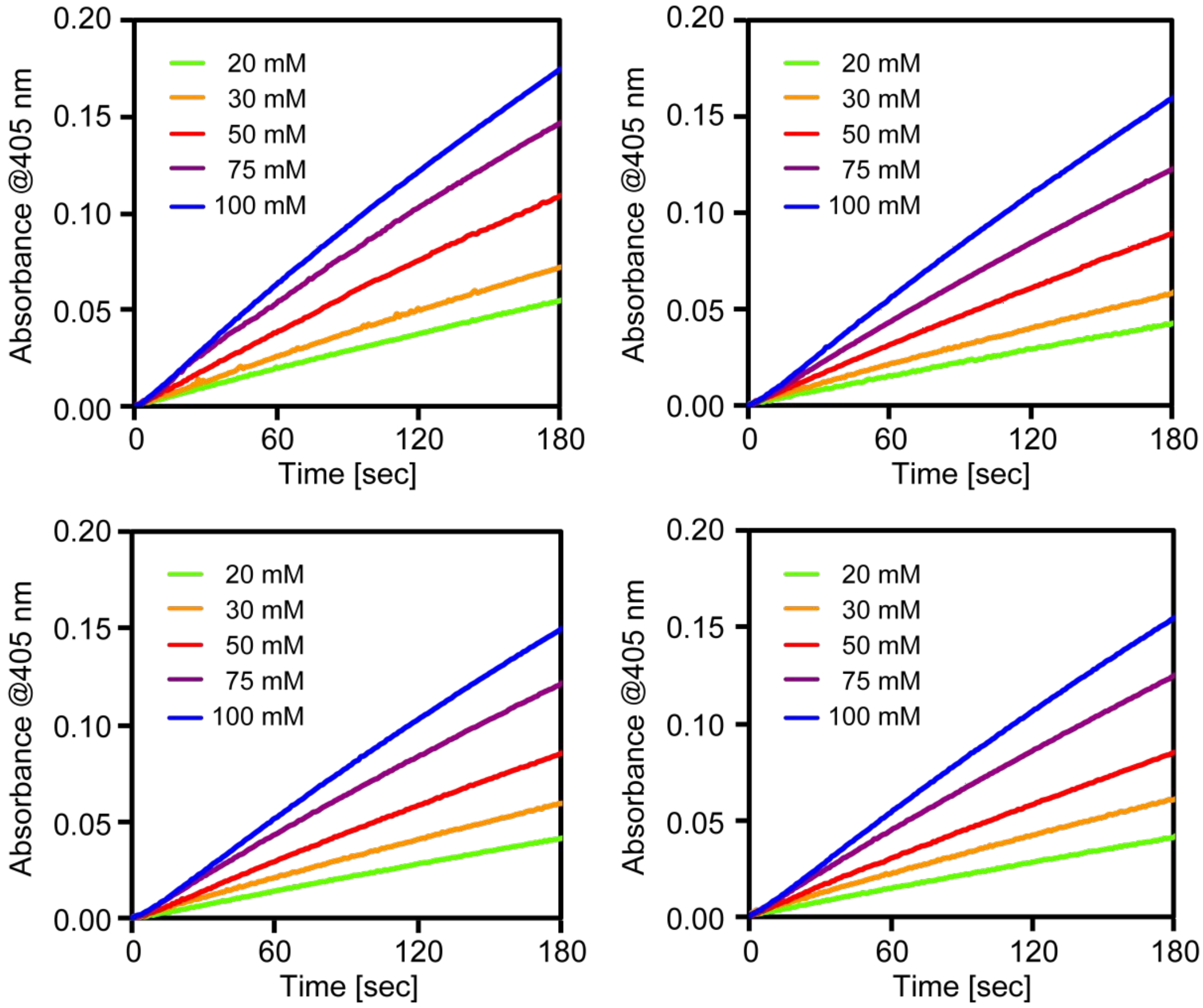

Figure S5. Time coarse data of absorbance at 405 nm contributed by the degradation of *p*-NPA with different concentrations: (a) native BSA, (b) PSAR_9-BSA, (c) PSAR_18-BSA, (d) PSAR_22-BSA (*n*=3).

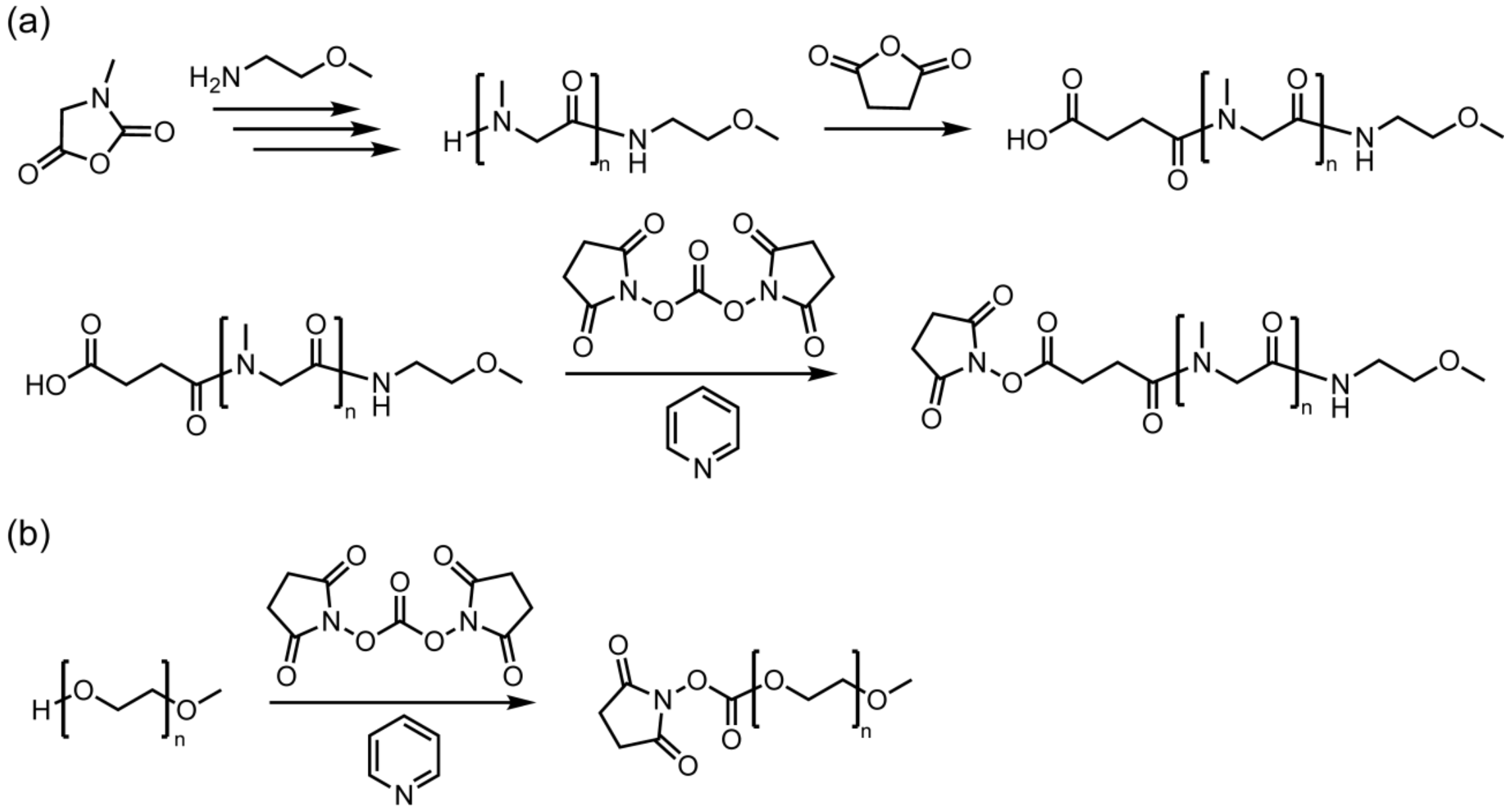

Scheme S1. Synthetic scheme of C-terminal activated mPSAR and mPEG.

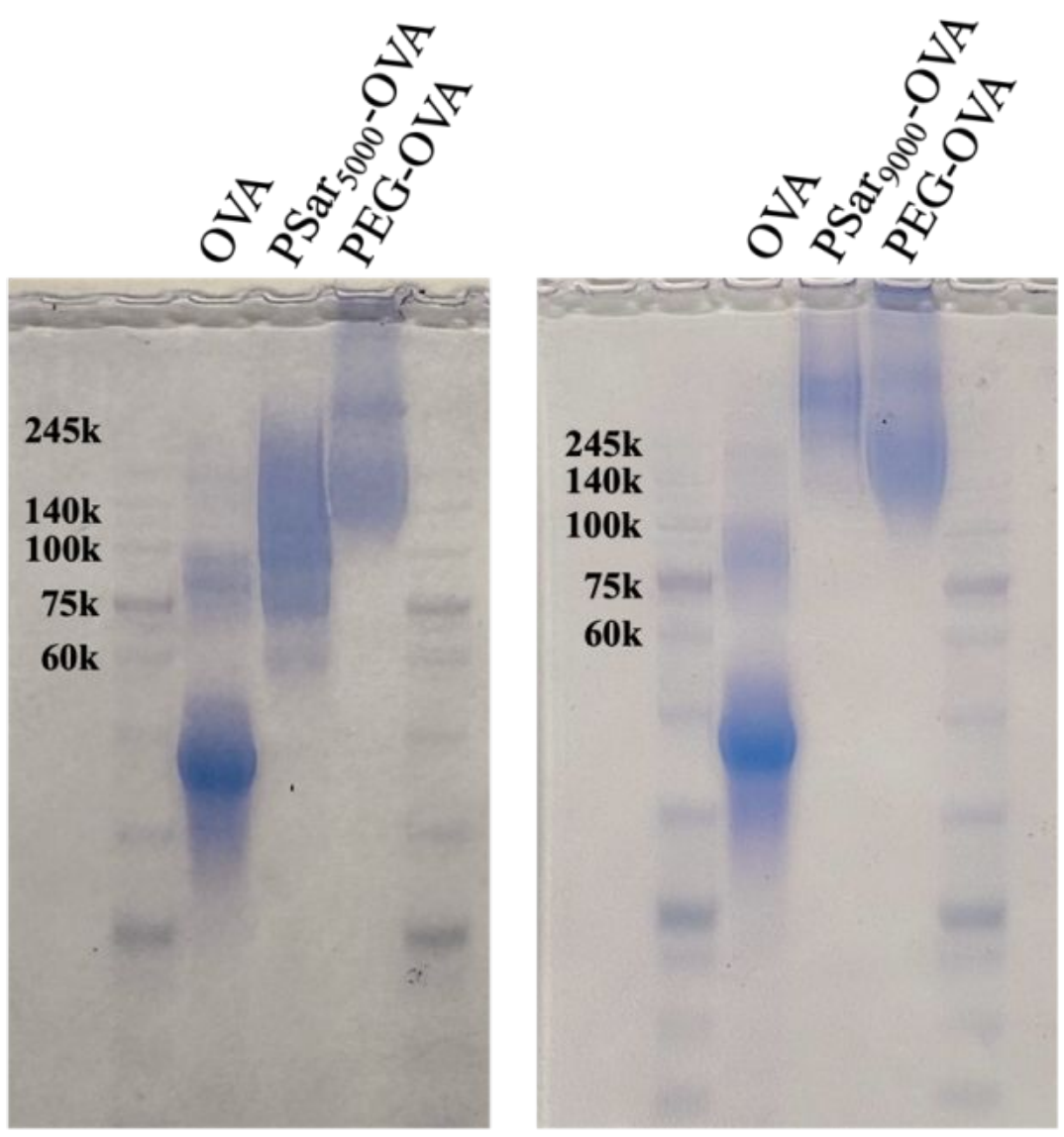

Figure S6. Molecular weights increase of OVA by the immobilization of mPSar and mPEG determined by SDS-PAGE.

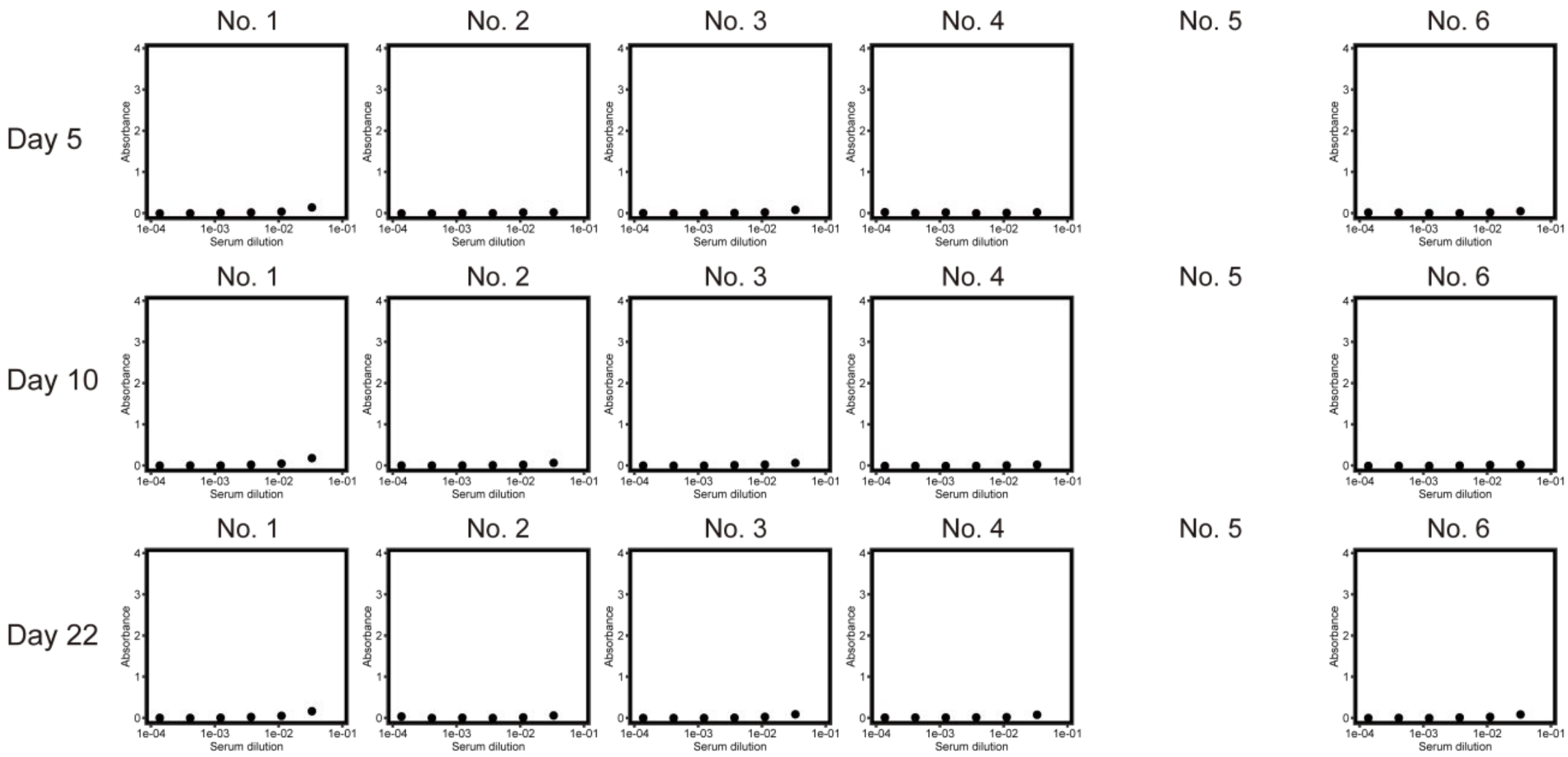

Figure S7. Evaluation of anti-poly(ethylene glycol) IgM titers in saline-dosed mice. Serum samples were collected from mice (*n* = 5) on the indicated days. Antibody reactivity was measured by enzyme-linked immunosorbent assay with poly(ethylene glycol) immobilized plates. The graphs show the absorbance (optical density) at 450 nm plotted against serial dilutions of the sera.

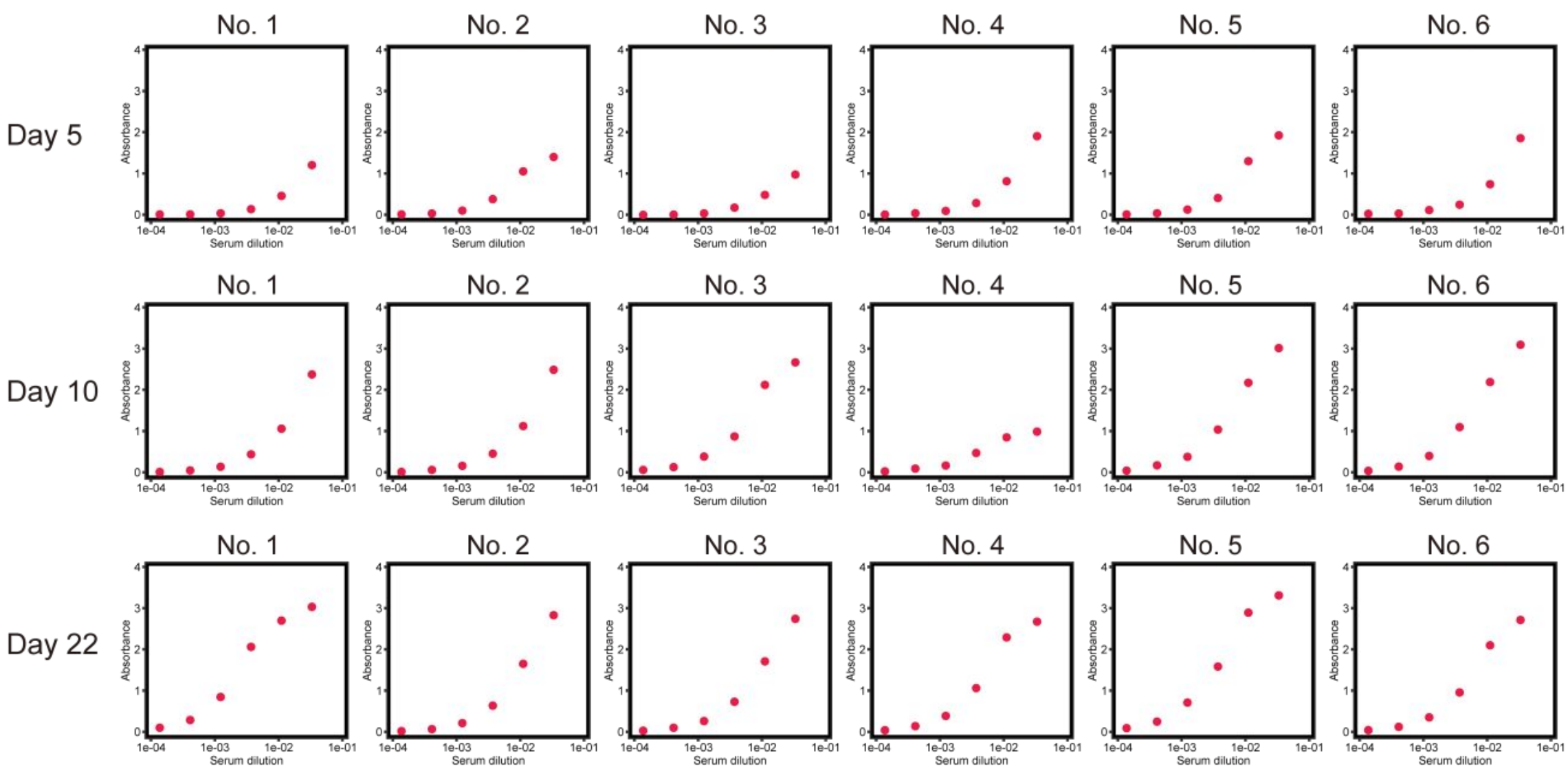

Figure S8. Evaluation of anti-poly(ethylene glycol) IgM titers in mPEG5k-OVA-dosed mice. Serum samples were collected from mice (*n* = 6) on the indicated days. Antibody reactivity was measured by enzyme-linked immunosorbent assay with poly(ethylene glycol) immobilized plates. The graphs show the absorbance (optical density) at 450 nm plotted against serial dilutions of the sera.

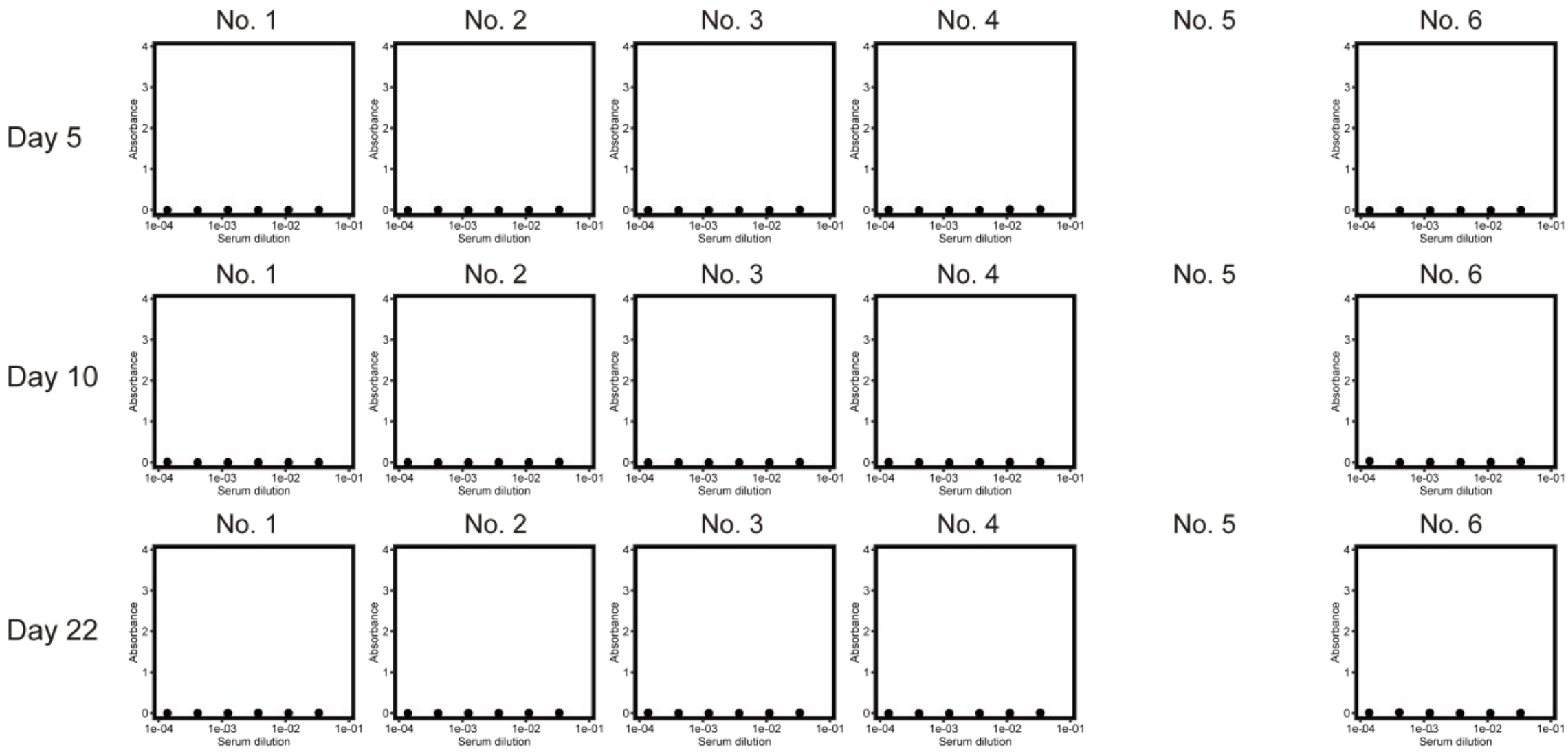

Figure S9. Evaluation of anti-poly(ethylene glycol) IgG titers in saline-dosed mice. Serum samples were collected from mice (*n* = 6) on the indicated days. Antibody reactivity was measured by enzyme-linked immunosorbent assay with poly(ethylene glycol) immobilized plates. The graphs show the absorbance (optical density) at 450 nm plotted against serial dilutions of the sera.

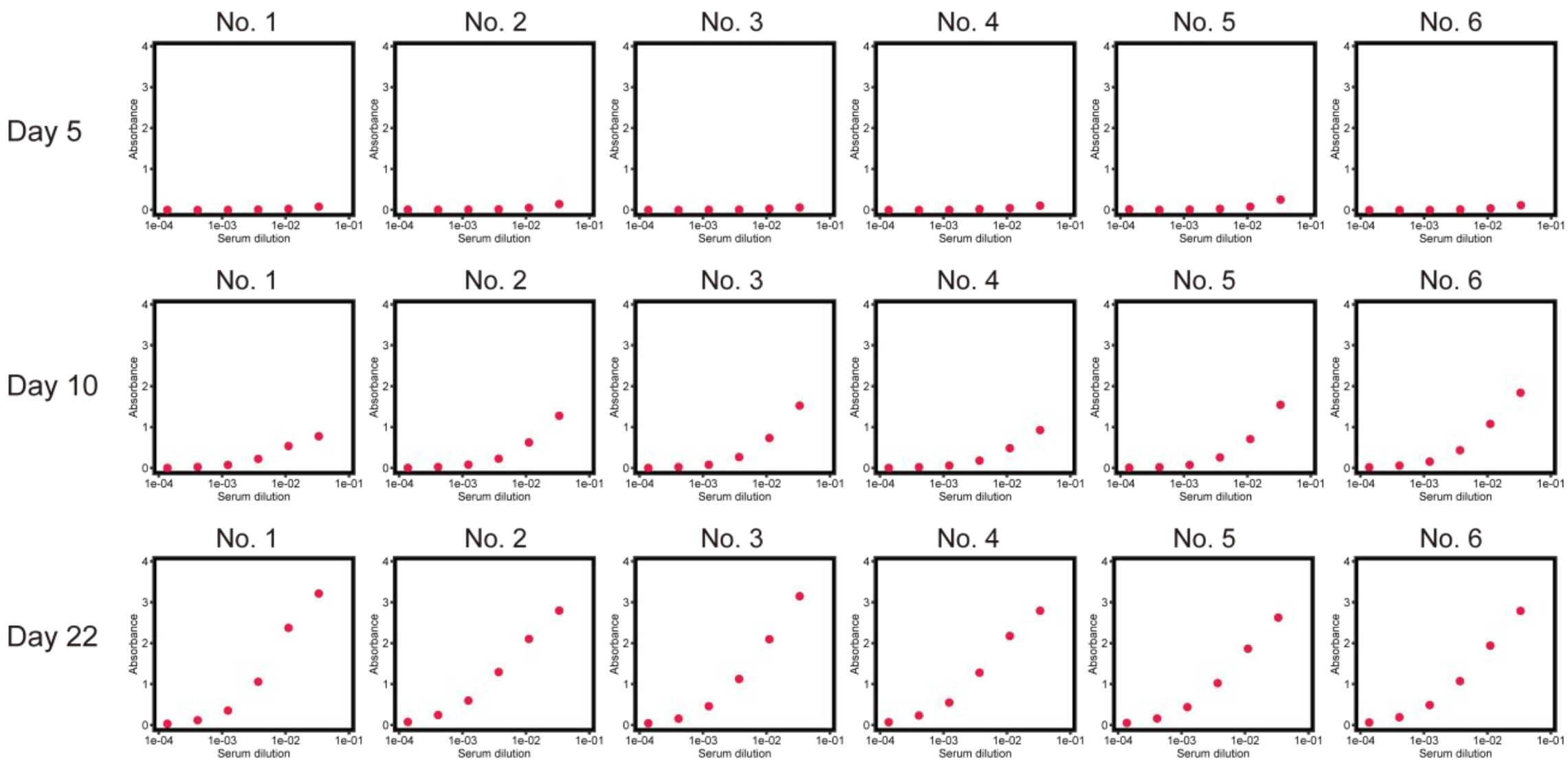

Figure S10. Evaluation of anti-poly(ethylene glycol) IgG titers in mPEG5k-OVA -dosed mice. Serum samples were collected from mice (*n* = 6) on the indicated days. Antibody reactivity was measured by enzyme-linked immunosorbent assay with poly(ethylene glycol) immobilized plates. The graphs show the absorbance (optical density) at 450 nm plotted against serial dilutions of the sera.

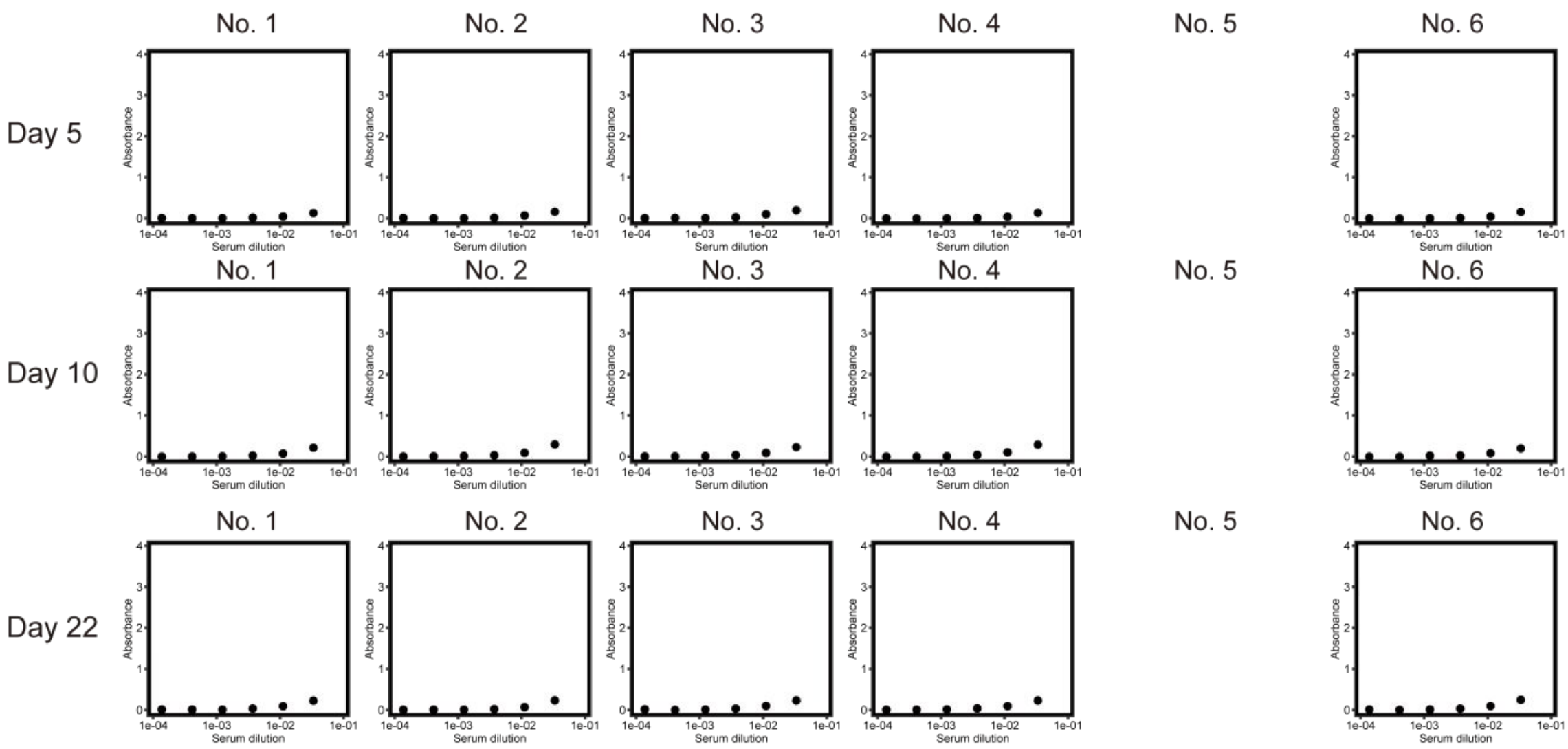

Figure S11. Evaluation of anti-poly(sarcosine) IgM titers in saline-dosed mice. Serum samples were collected from mice (*n* = 5) on the indicated days. Antibody reactivity was measured by enzyme-linked immunosorbent assay with poly(sarcosine) immobilized plates. The graphs show the absorbance (optical density) at 450 nm plotted against serial dilutions of the sera.

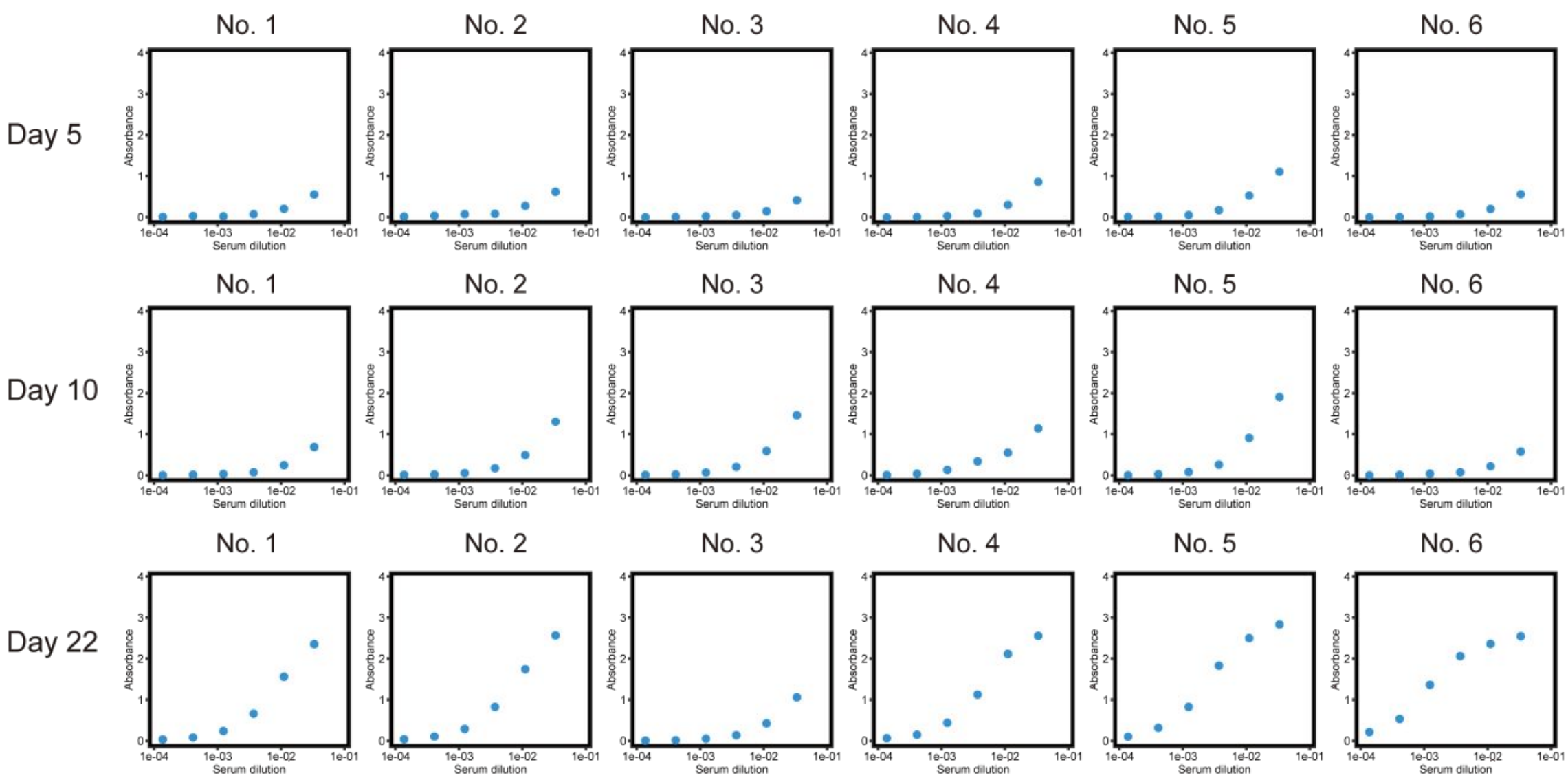

Figure S12. Evaluation of anti-poly(sarcosine) IgM titers in mPSAR5k-OVA -dosed mice. Serum samples were collected from mice (*n* = 6) on the indicated days. Antibody reactivity was measured by enzyme-linked immunosorbent assay with poly(sarcosine) immobilized plates. The graphs show the absorbance (optical density) at 450 nm plotted against serial dilutions of the sera.

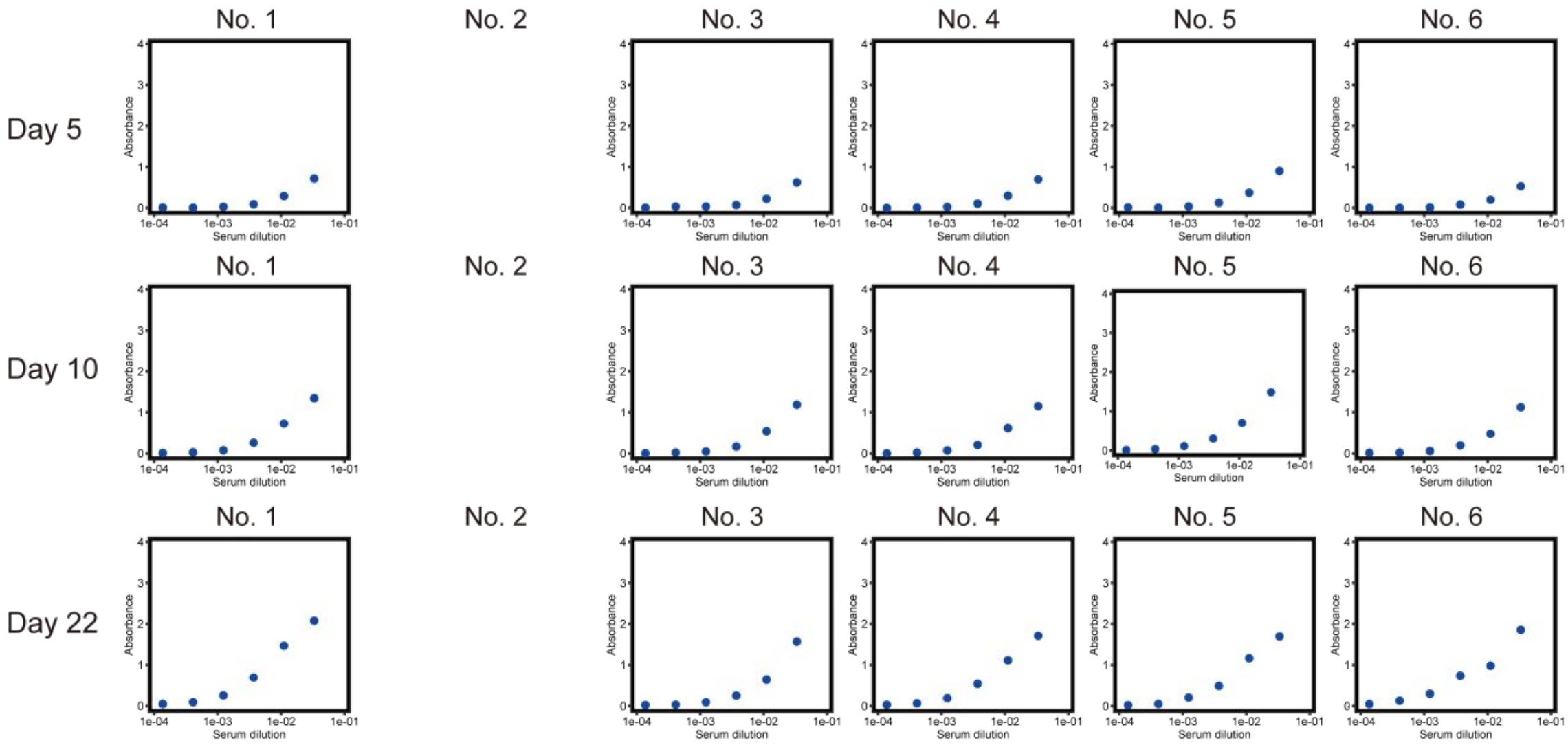

Figure S13. Evaluation of anti-poly(sarcosine) IgM titers in mPSAR9k-OVA -dosed mice. Serum samples were collected from mice (n = 5) on the indicated days. Antibody reactivity was measured by enzyme-linked immunosorbent assay with poly(sarcosine) immobilized plates. The graphs show the absorbance (optical density) at 450 nm plotted against serial dilutions of the sera.

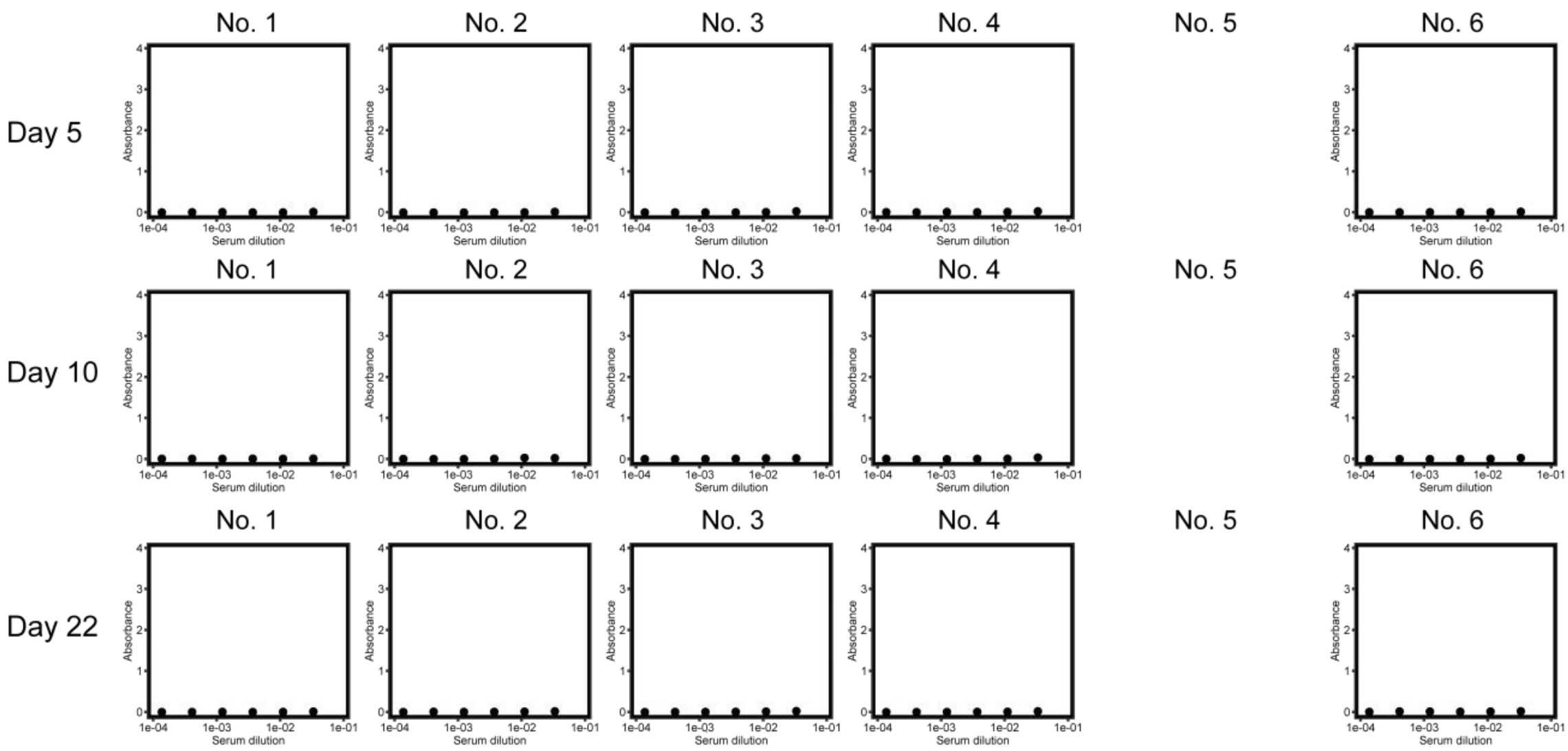

Figure S14. Evaluation of anti-poly(sarcosine) IgG titers in saline-dosed mice. Serum samples were collected from mice (n = 5) on the indicated days. Antibody reactivity was measured by enzyme-linked immunosorbent assay with poly(sarcosine) immobilized plates. The graphs show the absorbance (optical density) at 450 nm plotted against serial dilutions of the sera.

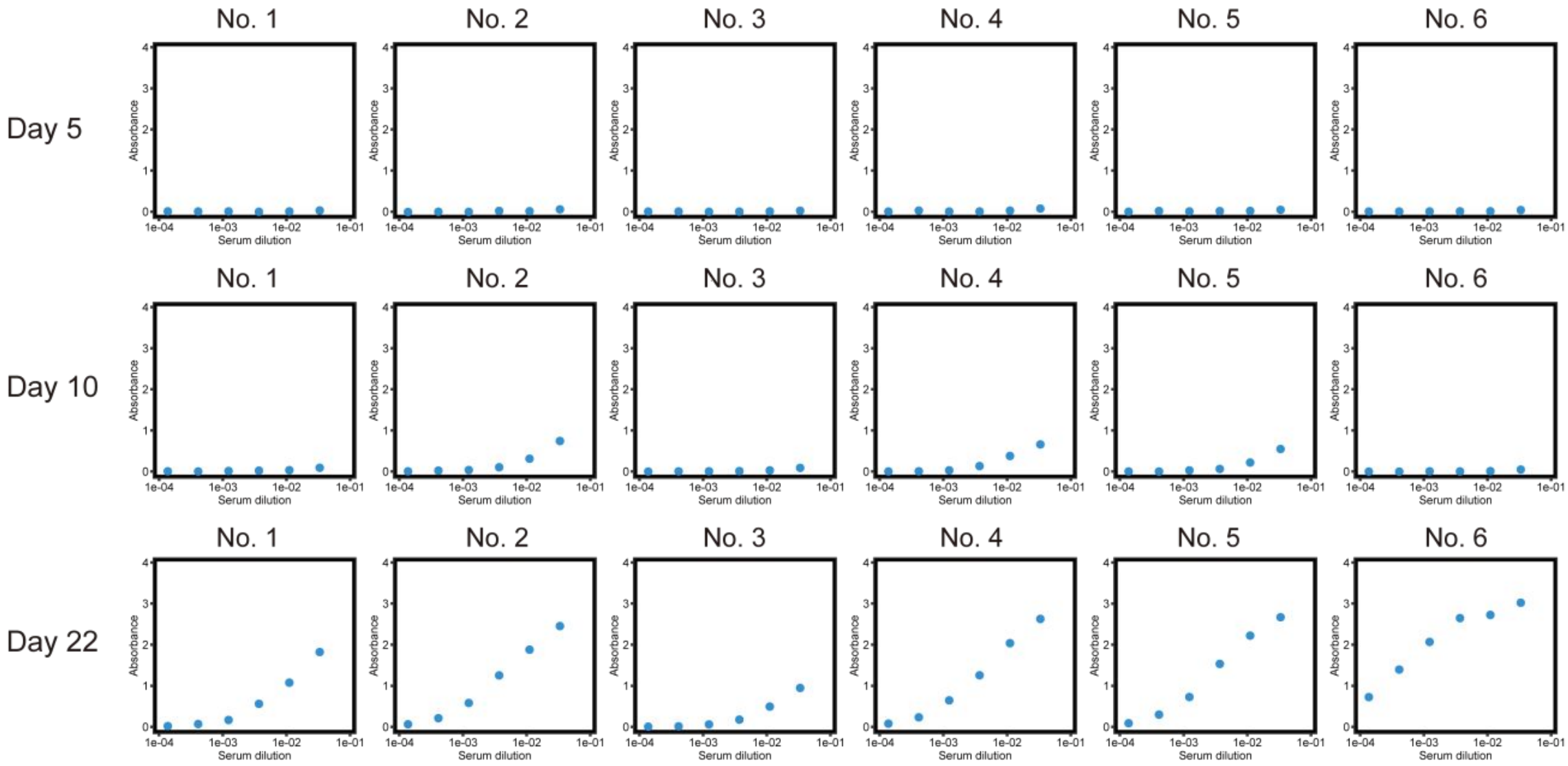

Figure S15. Evaluation of anti-poly(sarcosine) IgG titers in mPSAR5k-OVA-dosed mice. Serum samples were collected from mice (*n* = 5) on the indicated days. Antibody reactivity was measured by enzyme-linked immunosorbent assay with poly(sarcosine) immobilized plates. The graphs show the absorbance (optical density) at 450 nm plotted against serial dilutions of the sera.

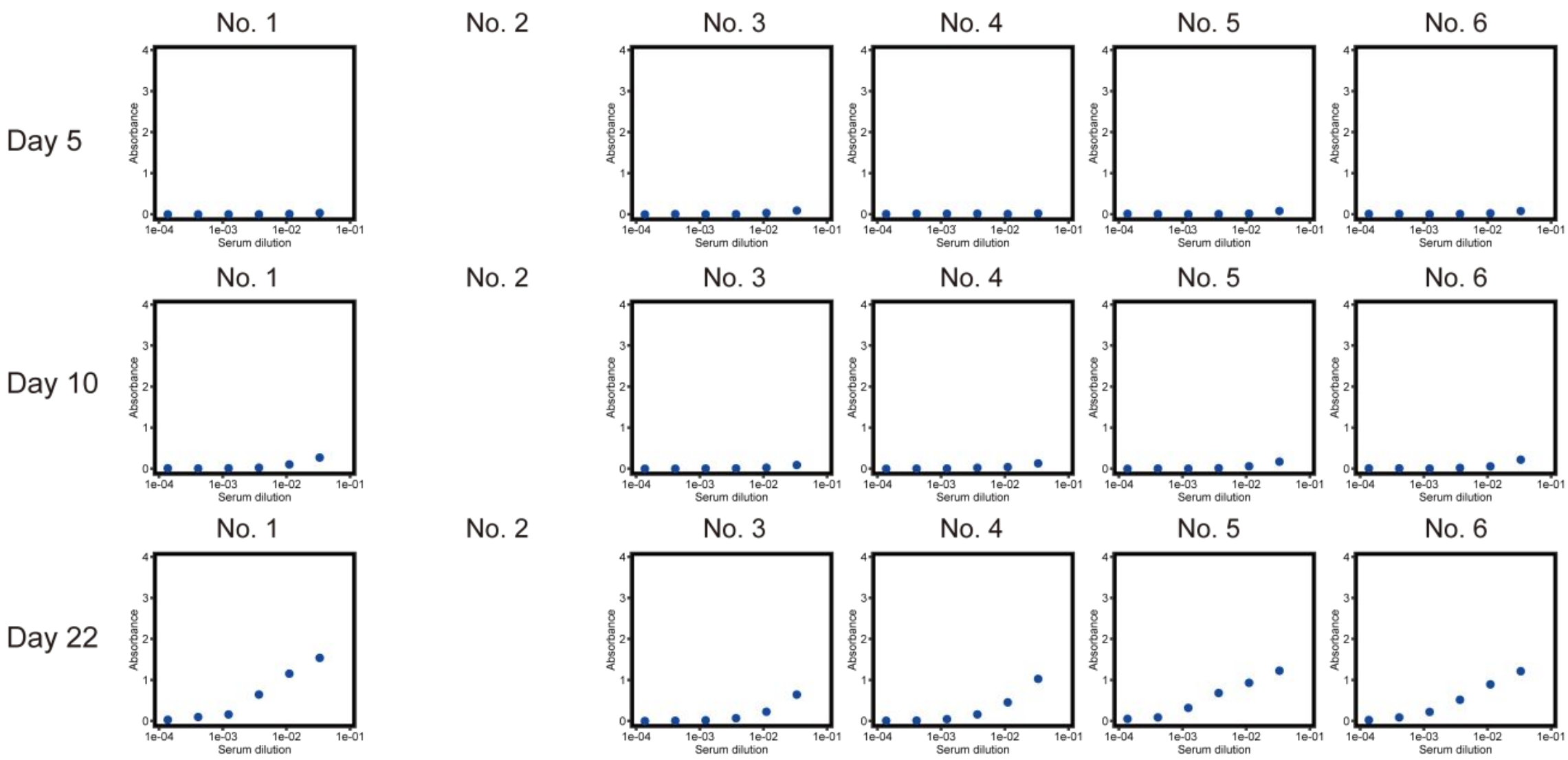

Figure S16. Evaluation of anti-poly(sarcosine) IgG titers in mPSAR9k-OVA-dosed mice. Serum samples were collected from mice (*n* = 5) on the indicated days. Antibody reactivity was measured by enzyme-linked immunosorbent assay with poly(sarcosine) immobilized plates. The graphs show the absorbance (optical density) at 450 nm plotted against serial dilutions of the sera.

References

(1) Schneider, C.A., Rasband, W.S., Eliceiri, K.W., NIH Image to ImageJ: 25 years of image analysis. *Nature Methods* **2012**, *9*, 671-675.

(2) Zhao, W.; Hu, J.; Gao, W. Glucose Oxidase–Polymer Nanogels for Synergistic Cancer-Starving and Oxidation Therapy. *ACS Appl. Mater. Interfaces* **2017**, *9* (28), 23528–23535. https://doi.org/10.1021/acsami.7b06814.
